# Optimal Icosahedral Copper-Based Bimetallic Clusters for the Selective Electrocatalytic CO_2_ Conversion to One Carbon Products

**DOI:** 10.3390/nano13010087

**Published:** 2022-12-24

**Authors:** Azeem Ghulam Nabi, Aman-ur-Rehman, Akhtar Hussain, Gregory A. Chass, Devis Di Tommaso

**Affiliations:** 1 Department of Chemistry, School of Physical and Chemical Sciences, Queen Mary University of London, Mile End Road, London E1 4NS, UK; 2 Department of Physics and Applied Mathematics, Pakistan Institute of Engineering and Applied Sciences, Nilore, Islamabad 45650, Pakistan; 3 Department of Physics, University of Gujrat, Jalalpur Jattan Road, Gujrat 50700, Pakistan; 4 Theoretical Physics Division, Pakistan Institute of Nuclear Science& Technology (PINSTECH), Nilore, Islamabad 45650, Pakistan; 5 Department of Nuclear Engineering, Pakistan Institute of Engineering & Applied Sciences, Nilore, Islamabad 45650, Pakistan; 6 Center for Mathematical Sciences, Pakistan Institute of Engineering & Applied Sciences, Nilore, Islamabad 45650, Pakistan; 7 Department of Chemistry, McMaster University, Hamilton, ON L8S 4L8, Canada; 8 Faculty of Land and Food Systems, The University of British Columbia, Vancouver, BC V6T1Z4, Canada

**Keywords:** CO_2_ reduction, copper catalysts, metal doping, density functional calculations

## Abstract

Electrochemical CO_2_ reduction reactions can lead to high value-added chemical and materials production while helping decrease anthropogenic CO_2_ emissions. Copper metal clusters can reduce CO_2_ to more than thirty different hydrocarbons and oxygenates yet they lack the required selectivity. We present a computational characterization of the role of nano-structuring and alloying in Cu-based catalysts on the activity and selectivity of CO_2_ reduction to generate the following one-carbon products: carbon monoxide (CO), formic acid (HCOOH), formaldehyde (H_2_C=O), methanol (CH_3_OH) and methane (CH_4_). The structures and energetics were determined for the adsorption, activation, and conversion of CO_2_ on monometallic and bimetallic (decorated and core@shell) 55-atom Cu-based clusters. The dopant metals considered were Ag, Cd, Pd, Pt, and Zn, located at different coordination sites. The relative binding strength of the intermediates were used to identify the optimal catalyst for the selective CO_2_ conversion to one-carbon products. It was discovered that single atom Cd or Zn doping is optimal for the conversion of CO_2_ to CO. The core@shell models with Ag, Pd and Pt provided higher selectivity for formic acid and formaldehyde. The Cu-Pt and Cu-Pd showed lowest overpotential for methane formation.

## 1. Introduction

The rising carbon dioxide (CO_2_) level and overall concentrations in the atmosphere due to fossil fuel combustion, a major cause of global warming, pose a serious threat to humankind [1]. One of the most promising solutions to mitigating this risk is via the chemical conversion of gaseous CO_2_ into value-added chemicals and materials [2]. The electrochemical CO_2_ reduction reaction (eCO_2_RR) has emerged as a potential strategy for converting CO_2_ because if coupled with electricity from renewable sources (wind, solar, or hydropower plants), the eCO_2_RR could achieve a carbon-neutral energy cycle [3,4]. The main challenges in eCO_2_RR lie in the activation of competitive CO_2_-minimizing pathways such as the hydrogen evolution reaction (HER, H^+^ + e^−^ → ½ H_2_) [5,6] and the conversion of CO_2_ to a specific product with good selectivity; this given the marginal difference in the electrochemical potentials of CO_2_ reduction into different products [3]. For example, the change for transforming CO_2_ to ethylene is −0.34 V while the CO_2_ to methanol transformation is −0.38 V, relative to the standard hydrogen electrode (SHE) [7].

Catalysts can facilitate favorable pathways to reduce the overall energy requirements of eCO_2_RR. Due to their ability to activate CO_2_, initial research focused on noble metal-based catalysts (Pt, Rh, Ir) [8,9,10,11,12], yet their scarcity and cost have retarded development. Hence, earth-abundant and active metal-based catalysts have been become requisite to develop sustainable solutions to CO_2_ transformation, when all aspects are considered (i.e., fundamental chemistry/physics, technological, economical). Copper (Cu) emerges as the best candidate for eCO_2_RR, being the only metal surface that reduces CO_2_ to more than thirty hydrocarbons and oxygenates [13], yet lacks the required selectivity [14,15,16]. Relevant studies dedicated to improving selectivity and hindering the HER have investigated the adsorption/desorption mechanism on single crystal Cu electrodes to demonstrate the role of surface morphology [17]. It was discovered that Cu crystal facets with high index planes such as Cu(711) are more selective in the production of valuable two-carbon (C2) products, such as ethylene and ethanol. This with respect to the dominant Cu(111) surface [18], while stepped Cu surfaces such as the (211) facet more easily produce one-carbon (C1) hydrocarbons [19]. Computational studies also revealed that the higher activity of polycrystalline Cu nanoparticles is due to the presence of stepped facets, such as (110) [20], (211) [21] and Cu(321) [22]. These stepped surfaces occur in metal clusters [18,22,23,24], where both the number of uncoordinated sites at the corners and edges [25] and the surface-to-volume ratio of nanoparticles are higher than those on copper surfaces, which may lead to improved catalytic properties towards eCO_2_RR [13].

Another strategy to improve the activity and selectivity of Cu electrodes is metal (M) doping [14]. Bimetallic catalysts often show better catalytic performance than the corresponding elemental metal ones due to synergic effects between the two metallic centers [26]. The dopant provides reaction sites with varied electronic properties and modulates those of the host (Cu), influencing the adsorption strength of the eCO_2_RR intermediates. Experimental studies have also revealed that low doping concentrations facilitate the formation of C1 products [27,28]. In particular, metal dopants such as Ag [28,29], Cd [30], Pd [28,31], Pt [32], and Zn [28,33] in Cu-M catalysts show efficiency towards C1/C2 products.

Consequently, in Cu-based catalysts, the nanoscale structuring and cooperative metal-metal coupling could enhance CO_2_ activation and selectivity, leading to specific product formation. In this regard, quantum mechanical modelling has provided insights into the structure, stability and catalytic properties of CuM clusters, while also demonstrating that an appropriate proportion of metal atoms influences the CO_2_ activation and selectivity towards the desired reaction. Alvarez-Garcia et al. investigated the binding and dissociation of CO_2_ on four-atom bimetallic Cu*_n_*Pd_4−*n*_ (*n* = 0–4) clusters employing density functional theory (DFT) calculations [30], resolving the ideal composition for adsorption energy and facile barriers to activation barrier was found in Cu_3_Pd; in agreement with the Pd/(Pd + Cu) atomic ratioing reported experimentally [34,35]. Investigation on the effect of substituting Cu with Zr on CO_2_ adsorption for the four-atom Cu_4_ cluster [36] revealed that the energy barriers for the direct dissociation of the CO_2_ molecule to CO + O decreased significantly for bimetallic CuZr clusters, with respect to pure Cu_4_. Our recent computational work on small tetrahedral CuSn clusters revealed the Cu_2_Sn_2_ system to suppress the competitive HER, while being highly selective towards the electrochemical CO_2_ → CO conversion [37]. Xing et al. considered bimetallic Pd*_n_*Cu*_m_* (*m* + *n* = 15, with *n* > *m*) clusters wherein Pd_10_Cu_5_ showed the highest catalytic activity, particularly towards the CO_2_ → COOH hydrogenation step [31]. Li et al. considered (Cu)*_n_* clusters with *n* = 8, 20, 38 (even numbers) and *n* = 13, 55 (odd numbers) to investigate the reactivity at the high density corner and edge sites and found the icosahedral Cu_55_ to provide the lowest energy pathway to the CO intermediate and the ensuing C2 ethylene product [25].

Computational characterizations of clusters in the size range of 10 ≤ *n* ≤ 55 showed that (Cu)*_n_* adopted the icosahedral structure [38] derived from 13- and 55-atom icosahedra, built by adding or removing atoms. In addition, a comparison of icosahedral and cuboctahedral (*n* = 55, 147 and 309) clusters confirmed the icosahedral copper clusters to be more stable. Experimental verification of the formation of copper clusters using microemulsion techniques revealed Cu_55_ to be one of the most abundant clusters followed by Cu_13_, Cu_147_ and Cu_309_ [39]. According to a recent DFT investigation, Cu_55_ exhibits highly degenerate states [40]; a direct outcome of its icosahedral symmetry. Therefore, study on nanoclusters such as the highly symmetric 55-atom icosahedral structures would give a deeper understanding than stepped surfaces. This has been attributed to their larger surface-to-volume ratio and higher proportion of coordinatively unsaturated surface atoms (corner or edge) in comparison to bulk materials, resulting in a narrowing of the d-band, an upward shift of the band’s energy, and consequently, a stronger adsorption of the reaction intermediates [41]. Investigation on the adsorption of CO_2_ on icosahedral 55-atom Cu-based bimetallic clusters [42] found that for the Cu_55−*x*_Zr*_x_* systems (*x* = 1–12), the formation of the CO_2_-activated state (linear to bent transition and elongation of C–O bonds) was endothermic on the pure copper cluster but barrierless and exothermic on the Zr-decorated system. Similarly, DFT calculations of Cu_55−*x*_Zr*_x_* systems (*x* = 0, 12, 13, 42, 43 and 55) with a core@shell and decorated distribution of Cu and Ni atoms showed the presence of Ni on the clusters was crucial to the activation of CO_2_ [43]. Although previous computational studies of icosahedral Cu-based bimetallic nanocatalysts considered the adsorption, activation and gas-phase dissociation of CO_2_, in the context of eCO_2_RR, the focus should be on the concerted proton-electron transfer (CPET) steps [44].

**Figure 1:**
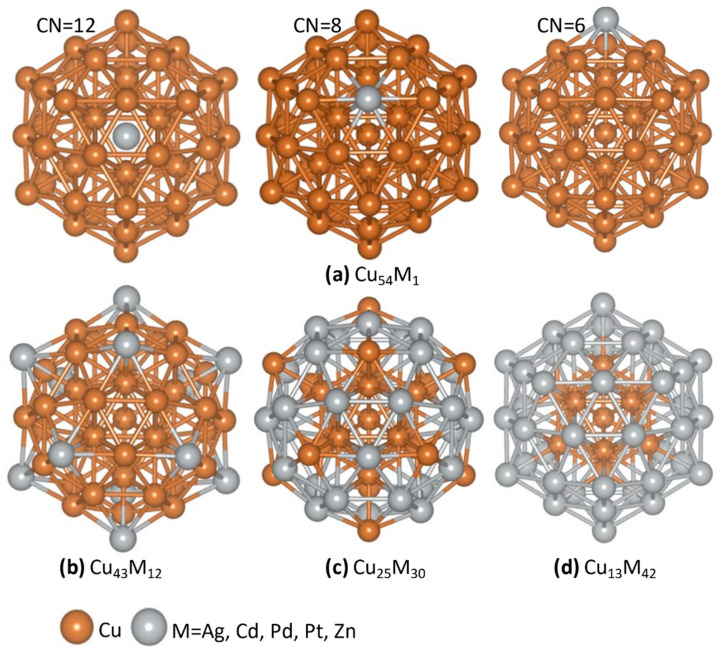
(**a**) The 55-atom Cu-based clusters doped with one metal atom (M) at three different coordination sites: CN = 6, 8 and 12. (**b**) The 55-atom Cu-based cluster doped with 12 metal atoms located at CN = 6. (**c**) The 55-atom Cu-based cluster doped with 30 metal atoms at CN = 8. (**d**) The core(Cu)@shell(M) cluster model.

Here, we present a computational investigation based on DFT calculations of the effect of nano-structuring and alloying in Cu-based catalysts on the activity and selectivity of the eCO_2_RR. Starting from the icosahedral Cu_55_ structure, we generated Cu_54_M_1_, Cu_43_M_12_ and Cu_30_M_25_ decorated architectures and Cu_13_M_42_ core@shell models (M = Ag, Cd, Pd, Pt, and Zn) (Figure 1), with the metals located at three different coordination sites (6, 8 and 12). We provide a thorough analysis of the structural, thermodynamic and electronic properties of these nanoclusters and their ability to activate CO_2_. The computational hydrogen electrode (CHE) model [45] was then applied to compute the mechanism of eCO_2_RR to the C1 products carbon monoxide (CO), formic acid (HCOOH), formaldehyde (CH_2_O), methane (CH_4_) and methanol (CH_3_OH). We have focused our attention to C1 products because a recent techno-economic assessment of low-temperature CO_2_ electrolysis shows the production costs of C1 products such as HCOOH and CO are competitive to conventional processes compared to C2 products such as ethylene and ethanol, which production has substantially higher costs [46] We compare the free energy profiles for the electrocatalytic CO_2_ conversion to these C1 products to the competitive HER. The relative binding strength of the intermediates involved is used to identify catalysts for the selective CO_2_ conversion. For comparison purposes, calculations of the eCO_2_RR and HER were also conducted on the (100), (110), (111) and (211) facets of pure copper.

## 2. Computational Methods

### 2.1. Atomistic Models of Clusters and Surfaces

The icosahedral (I_h_) 55-atom monometallic Cu cluster was generated using the ab initio random structure searching (AIRSS) code [47]. The decorated Cu_54_M clusters were then generated by replacing one surface Cu with a dopant metal atom M, where M = Ag, Cd, Pd, Pt and Zn. As shown in Figure 1a, there are three possible coordination sites: CN6 is the edge site, CN8 is the corner site and CN12 is the center of the nanocluster. The Cu_43_M_12_ model in Figure 1b was generated by replacing 12 Cu atoms with M located at CN6. The Cu_25_M_30_ model in Figure 1c was generated by replacing 12 Cu atoms with M located at CN8. The Cu_13_M_42_ core@shell model in Figure 1d was generated by replacing all 13 surface Cu atoms with M. We also considered four-layer (3 × 3) slab models of Cu(100), Cu(110), Cu(111) and Cu(211) [20] with the (100), (110) and (111) being the dominant surfaces of copper. The Cu(211) facet was considered because of its good selectivity towards C1 formation. This was linked to the Cu(211) morphology characterized by step-edge sites with a coordination number equal to 7 (CN7) [48]. Here, we have also compared the catalytic conversion of CO_2_ to C1 chemicals on Cu(211) to that on 55-atoms icosahedral Cu-M nanoclusters with M located at CN6 and CN8.

### 2.2. Density Functional Theory Calculations

Calculations of energies and structures were conducted at the spin-polarized DFT level using the “Vienna ab initio simulation package” (VASP Software GmbH, version 6.3.1, Vienna, Austria) [49] using the following computational settings: the Perdew–Burke–Ernzerhof (PBE) exchange correlation functional with the Grimme’s-D3 dispersion correction; a plane-wave basis set within the framework of the projector augmented wave method with a kinetic energy cutoff (*E*_cut_) set to 400 eV; a single *k*-point (1 × 1 × 1) for the nanoclusters and a (5 × 5 × 1) *k*-point mesh for the surface model to sample the Brillouin zone of the simulation supercell; a 0.18 eV width for the smearing. Energies, zero-point energies, and entropies of H_2_(g), CO_2_(g) and CO(g), and H_2_O used to compute the free energy corrections are reported in Supplementary Information (Table S1).

### 2.3. Free Energy Calculations

Following the computational hydrogen electrode (CHE) method proposed by Nørskov and co-workers [45], the Gibbs free energy of each step involved in the eCO_2_RR to C1 products was computed using the following equation:

(1) $$\Delta G=\Delta S+\Delta E_{\mathrm{ZPE}}-T\Delta S+\Delta G_{\mathrm{solv}}+\Delta G_{U}$$

where Δ*E* is the reaction energy; Δ*E*_ZPE_ is the change in zero-point energy; Δ*S* is the change in entropy and *T* is the temperature of the reaction (300 K). We determined the latter two quantities within the harmonic approximation by taking the vibrational frequencies of adsorbates and molecules calculated with DFT. The solvation effects to compute the solvation free energy term Δ*G*_solv_ were included using VASPsol [50]. Δ*G*_U_ is the free energy correction introduced by the difference of the electrode potential. For reactions involving a concerted proton–electron transfer (CPET) step, the Δ*G*_U_ term can be computed by applying the formula:

(2) $$\Delta G_{U}=-neU$$

where *n* is the number of electrons transferred, *e* is the electron charge and *U* is the applied electrode potential. The limiting potential (*U*_L_) and the overpotential (*η*) are important factors for evaluating the catalytic activity. The limiting potential is given by the formula:

(3) $$U_{L}=-\Delta G_{\max}/ne$$

where ∆*G*_max_ is the relative change of the Gibbs free energy of the rate-determining step. The overpotential (*η*) can be obtained by calculating the difference between the equilibrium potential (*U*_eq_) and the limiting potential:

(4) $$\eta =U_{\mathrm{eq}}-U_{L}$$

Thus, *η* represents the minimum applied potential required to facilitate the formation of relevant intermediates.

## 3. Results and Discussion

### 3.1. Stability, Structure, and Electronic Properties of the Icosahedral 55-Atom CuM Clusters

**Figure 2:**
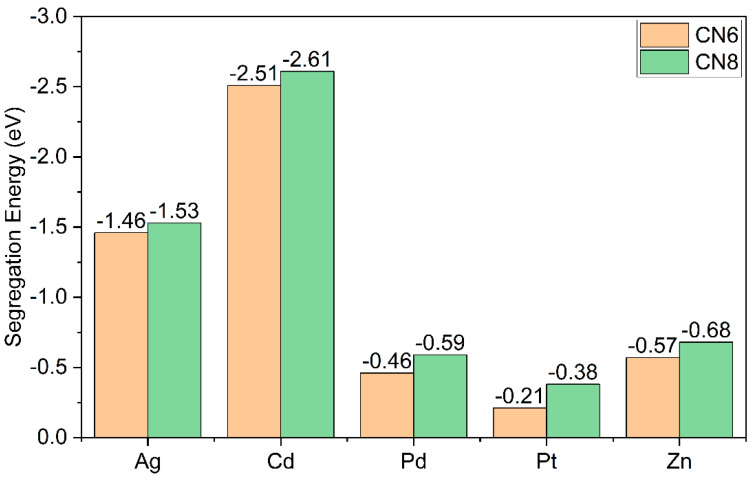
The segregation energy of the metal dopants Ag, Cd, Pd, Pt, and Zn in Cu_54_M clusters. Dopant metal located at two different coordination sites: CN6 and CN8.

The segregation energy (*SE*) was used to determine the preference of the metal dopants (Ag, Cd, Pd, Pt and Zn) to be in the core or shell of Cu_54_M. The *SE* is defined as [51]:

(5) $$SE=E\left[{\mathrm{Cu}}_{54}M\left(\mathrm{surface}\right)\right]-E\left[{\mathrm{Cu}}_{54}M\left(\mathrm{core}\right)\right]$$

where *E*[Cu_54_M(surface)] and *E*[Cu_54_M(core)] are the electronic energies of the fully optimized Cu_54_M_1_ cluster obtained by replacing one Cu atom with a dopant metal at a surface (CN6 or CN8) and at the center of the cluster (CN12), respectively. In Figure 2, the *SE* values are negative for all Cu_54_M, which implies that the metal prefers to be at the surface of the cluster, consistent with DFT calculations of Cu_54_Zr [51]. The metal doping at the CN8 site is more stable than CN6, but because their values of *SE* were close, the adsorption and reduction of CO_2_ on both coordination sites.

To gain insights into the relative stability of pure and bimetallic 55-atom systems, we used the binding energy per atom (*E*_B_), defined as [52]:

(6) $$E_{B}=\frac{E\left({\mathrm{Cu}}_{55-x}M_{x}\right)-\left(55-x\right)E\left(\mathrm{Cu}\right)-xE\left(M\right)}{55}$$

where *E*(Cu_55−*x*_M*_n_*) is the total energy of the most stable isomer of each Cu_55−*x*_M*_x_* cluster and *E*(Cu) and *E*(M) are the total energies of the Cu and Sn atoms, respectively. A higher negative value of *E*_B_ indicates higher thermodynamic stability of the cluster. The calculated *E*_B_ for pure Cu_55_ nanocluster is –2.99 eV, equal to the value obtained using all-electron triple-z quality DFT calculations [53]. Table 1 reports the calculated *E*_B_ and other structural and electronic properties: the average interatomic bonding distance between nearest neighbors, the energy difference between the highest occupied molecular orbital (HOMO), the lowest unoccupied molecular orbital (LUMO) (Δ_H−L_), the Bader charge difference between the Cu and M atoms (∆Q_M_), and the surface energy (*γ*). The surface energy was computed using the following equation [54]:

(7) $$\gamma =\frac{E_{nanosphere\;}-NE_{bulk}}{4\pi R^{2}}\;$$

where *E*_nanosphere_ is the energy of a cluster with *N* atoms (*N* = 55), *E*_bulk_ is the energy of the bulk material per one layer of cross-section and *R* is the radius a spherical incorporating the cluster.

A descriptor to analyze the global reactivity descriptor is the gap energy Δ_H−L_, which relates to the energy cost for an electron to jump from the HOMO to the LUMO orbital. Therefore, Δ_H−L_ characterizes the chemical stability of the system, with a higher value corresponding to a more chemically stable (less reactive) cluster. The Δ_H−L_ for pure Cu_55_ atom is 0.028 eV, consistent with the literature value of 0.03 eV [55]. In Table 1 and Figure S1b of Supplementary Information, the single-doped atom clusters Cu_54_Ag (CN6) and Cu_54_Zn (CN6) show small Δ_H−L_ values of 1 × 10^−4^ and 5 × 10^−4^ eV, respectively. The single atom doped Cu_54_Ag (CN8) and Cu_54_Zn (CN8) show larger Δ_H−L_ values of 4.9 × 10^−2^ and 4.1 × 10^−2^ eV. This shows that the coordination environment of the metal dopant affects the gap energy Δ_H−L_ and, therefore, the reactivity of the cluster. Overall, the values of Δ**_H−L_** are between 1 × 10^−4^ and 1.7 × 10^−1^ eV. The highest value of Δ_H−L_, 1.7 × 10^−1^ eV, is found for Cu_43_Zn_12_. The charge distribution in CuM clusters depends on the doping metal. This will influence CO_2_ adsorption and subsequent CO_2_ reduction because the electron transfer occurs from the electron-rich metal to the C atom, which in CO_2_ is in its highest oxidation state. In the decorated clusters (Cu_54_M, Cu_43_M_12_, and Cu_25_M_30_), when M is Ag, Pd or Pt, charge is transferred from Cu to M (negative ∆Q_M_), and when M is Cd or Zn charge is transferred from M to Cu (positive ∆Q_M_). In the core@shell Cu_13_M_42_ architecture, when M = Ag, Pd and Pt, the core is positively charged because of the charge transfer from Cu to M, while the shell has a negative charge. Vice versa for Cu_13_M_42_ with M = Cd and Zn. The effect of atomic radii, covalent radii, van-der radii, and electronegativity difference *(∆E*_N_) on bond lengths and surface area are discussed and available in Supplementary Information (Table S2). Regarding the surface energy, all clusters have negative γ values signifying the stability of the clusters compared to the bulk.

### 3.2. Adsorption and Activation of CO_2_ on Cu and CuM Clusters

**Figure 3:**
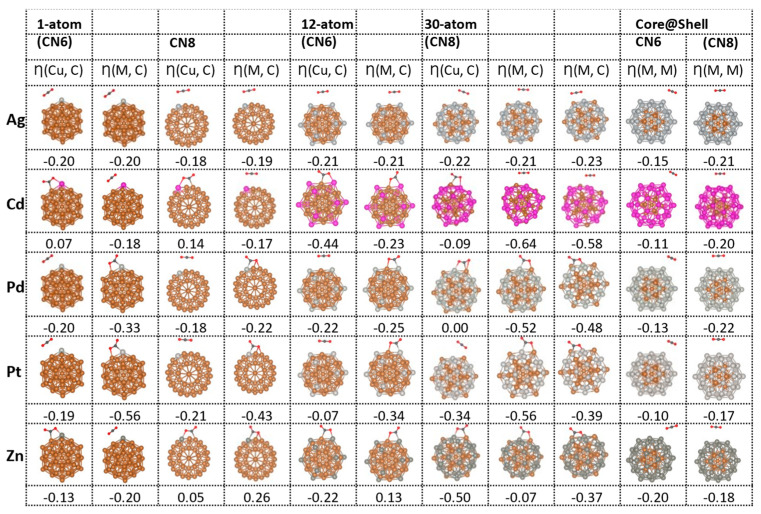
The structures and adsorption energies (in eV) of CO_2_ on the CuM clusters.

CO_2_ is a linear molecule with two equivalent C–O bonds (length = 1.12 Å).^13^ Before its dissociation, the first step in the catalytic conversion of CO_2_ is its adsorption on the catalyst surface. CO_2_ can maintain the geometric properties of gas-phase CO_2_ (physisorption) or become activated because of the charge transferred from the metal catalyst to the π* molecular orbitals of the CO_2_ molecule (chemisorption) resulting in the elongation of the C–O bonds and decrease in the O–C–O bond angle (linear to bent mode) [56]. Here, we have conducted a detailed characterization of the adsorption and activation of CO_2_ on the pure copper cluster Cu_55_ and the copper-metal clusters, Cu_54_M (CN6 and CN8), Cu_43_M_12_ (CN6), Cu_25_M_30_ (CN8) and Cu_13_M_42_ (core@shell), with M = Ag, Cd, Pd, Pt and Zn. These models can provide insights into the influence of surface chemistry on the activation of the CO_2_ molecule. The structures of CO_2_ on the CuM clusters are shown in Figure 3. The associated values of adsorption energies (*E*_ads_), bond angles, bond elongations and Bader charges of CO_2_ adsorbed on the CuM clusters are listed in Table 2. The adsorption energy was calculated as

(8) $$E_{ads}=E_{CuM\cdot \cdot \cdot CO_{2}}-E_{CuM}-E_{CO_{2}}$$

where the first term is the total energy of the CuM···CO_2_ system, and the second and third terms are the energies of the isolated cluster and CO_2_ molecules, respectively. CO_2_ is physisorbed on all Cu-Ag and core@shell clusters as indicated by the absence of significant deviations of the bond angle, bond elongation of adsorbed CO_2_ from the gas-phase values, and small charge transfer between Cu and M (*∆*Q_M_ ~0.04e). In Figure 3 and Table 2, η(Cu, C) and η(M, C) refer to configurations in which the C atom of the CO_2_ molecule is coordinated to the Cu and M atoms, respectively. In each chemisorbed state, there is a decrease in the O–C–O angle and an increase in charge transfer. In the single metal-doped systems, Cu_54_M, at the CN6 active site, the coordination state η(Cu, C) occurs for M = Cd and Zn and η(M, C) occurs for M = Pd and Pt. In single metal-doped clusters at CN8, the η(Cu, C) exists for M = Cd and η(M, C) is present for M = Pd and Pt. Both η(Cu, C) and η(Zn, C) exist for Cu_54_Zn on the CN8 active site. In the 12-atom doped Cu_43_M_12_ clusters, η(Cu, C) is present for M = Cd, Zn and η(M, C) exists in all systems except for Cu_43_Ag_12_. The 30-atom doped nano catalysts show the same trend as the 12-atom, except for the absence of η(Cd, C) in Cu_43_Cd_12_. The η(Cu, C) and η(M, C) coordination do not exist in the core@shell models because CO_2_ only physisorbs. In terms of adsorption energy, in the absence of η(M, C), the physisorption energy of CO_2_ always dominates. Similarly, in the absence of η(Cu, C), the chemisorption energy of η(M, C) configuration always dominates. When both η(Cu, C) and η(M, C) occur on a particular site, then again η(Cu, C) is the most stable coordination mode.

### 3.3. Mechanism of CO_2_ Reduction Reaction to C1 Products on Cu-M Clusters and Cu Surfaces

**Scheme 1:**
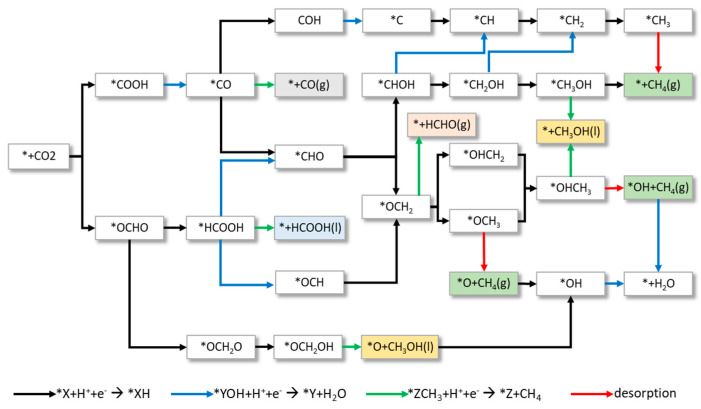
Reaction pathways to the C1 products CO, HCOOH, CH_2_O, CH_4_ and CH_3_OH.

In this section, we present calculations of the mechanism of electrochemical CO_2_ reduction. Scheme 1 shows the pathways and intermediates for the formation of the following C1 products: CO, HCOOH, CH_2_O, CH_4_ and CH_3_OH. Depending on the atom coordinated to the catalyst, O or C, the first CPET step leads to two intermediates, *OCHO and *COOH. The second CPET will determine whether the 2e^−^ products HCOOH or CO is formed. Subsequent CPET will lead to 4e^−^ (CH_2_O), 6e^−^ (CH_3_OH) and 8e^−^ (CH_4_) C1 products. Compared to other catalytic reactions, the pathway of the eCO_2_RR is more complex because of the number of intermediates involved. According to Equation 1, the optimal reaction pathway is determined by the lowest free energy pathway at the applied potential *U.*

#### 3.3.1. Electrocatalytic CO_2_ conversion to CO and HCOOH

We computed the free energy of reactions (Δ*G*) of the elementary steps to the CO_2_ conversion to HCOOH and CO on the following systems: icosahedral Cu_55_ cluster; decorated and core@shell Cu-M bimetallic clusters; Cu(100), Cu(110), Cu(111) and Cu(211) surfaces. In the context of the CHE model (see Equation 1), we define the potential limiting step (Δ*G*_PLS_) as the elementary reaction in the eCO_2_RR to CO or HCOOH (at *U* = 0 V) with the highest Δ*G* value (a high Δ*G*_PLS_ corresponds to poor catalytic performance). The elementary steps leading to the formation of CO are: (i) CO_2_ adsorption (CO_2_ → *CO_2_, ΔG_*CO2_); (ii) CPET to convert *CO_2_ to C-coordinated formate (*CO_2_ + H^+^ + e^−^ → *COOH, ΔG_*COOH_); (iii) CPET to convert formate to adsorbed carbon monoxide (*COOH + H^+^ + e^−^ → *CO + H_2_O, ΔG_*CO_); (iv) the release, from the catalyst surface, of gas-phase CO (*CO → CO(g), Δ*G*_CO_). The structures of the optimized *COOH and *CO on all Cu and CuM systems are reported in Supplementary Information (Figures S2–S8).

**Figure 4:**
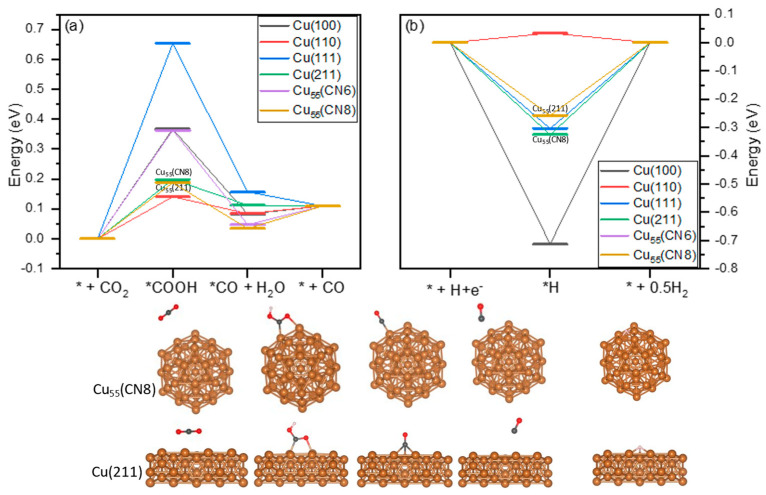
The Gibbs free energy diagram for the (**a**) CO_2_ reduction reaction to CO and the (**b**) hydrogen evolution reaction on Cu_55_ cluster and on the (100), (110) and (111) Cu surfaces.

**Pure copper: CO_2_-to-CO conversion.** The Gibbs free energy diagrams for the CO_2_-to-CO conversion on the monometallic 55-atom cluster and the (100), (110), (111) and (211) surfaces are reported in Figure 4a. There is a significant dependence of the stability of the *COOH and *CO intermediates on the surface morphology and coordination environment. The (211) facet has better catalytic performance (lower Δ*G*_PLS_) towards CO formation than any other surfaces but higher than Cu_55_. This cluster was, therefore, taken as the reference system to assess the performance of the bimetallic clusters. The competitive HER (H^+^ + e^−^ → ½ H_2_) in Figure 4b shows a similar morphology dependence: unfavourable on the (110) surface; highly favorable on the (100) surface; moderately favorable on the (211) surface and the Cu_55_ cluster.

**Figure 5:**
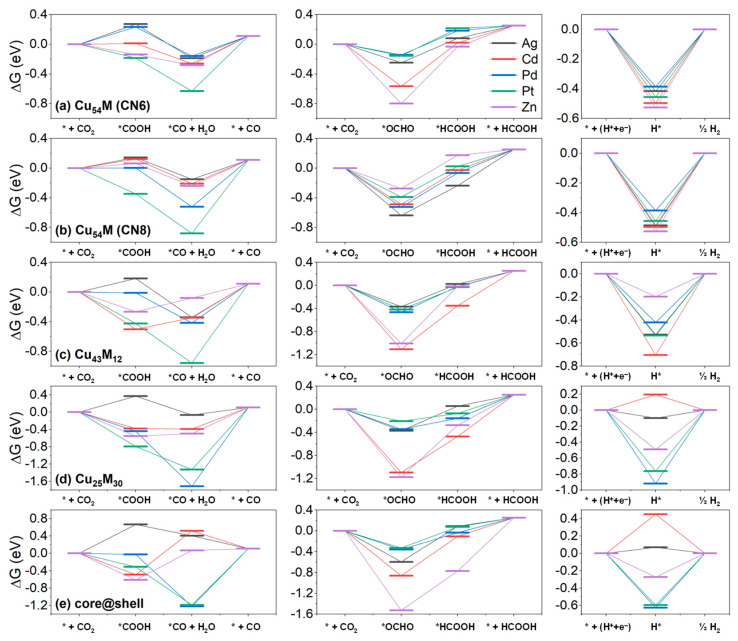
The Gibbs free energy diagram for the CO_2_ reduction to CO (left), CO_2_ reduction to HCOOH (centre), and hydrogen evolution reaction (right) on the CuM clusters (M = Ag, Cd, Pd, Pt and Zn): (**a**) Cu_54_M with CN6, (**b**) Cu_54_M with CN8, (**c**) Cu_43_M_12_, (**d**) Cu_25_M_30_ and (**e**) core@shell.

**Bimetallic clusters: CO_2_-to-CO conversion.** The free energy diagrams for the eCO_2_RR to CO and the HER on CuM are reported in Figure 5. In the single metal doped clusters, Cu_54_M, the value of ΔG_PLS_ depends on both the coordination site and nature of the metal. The Δ*G*_PLS_ is lower when the reaction occurs on CN6 for M equal to Cd (0.16 eV), Pd (0.23 eV) and Pt (0.53 eV) compared to CN8, Cd (0.12 eV), Pd (0.42 eV) and Pt (0.78 eV). However, for Ag (0.27 eV) and Zn (0.18 eV), CN6 shows higher Δ*G*_PLS_ than CN8, Ag (0.14 eV) and Zn (0.14 eV). Each intermediate shows strong chemisorption with a low ΔG_PLS_ value and vice versa. For both CN6 and CN8 systems, HER is dominant (lower ΔG_H_) over eCO_2_RR because of the strong CO binding to the cluster, leading to a large ΔG_*CO_; the exception is M = Pt. When the number of metal dopants on the CuM cluster increases, Cu_43_M_12_, so does the value of ΔG_PLS_: Ag (0.24 eV), Pd (0.32 eV), Pt (0.86 eV) and Zn (0.19 eV). The exception is Cd (0.25 eV). The CO generation remains dominant over HER, except again for Pt, for the same reasons discussed for the single atom doped clusters. With further increase in the doping and change in surface chemistry in the Cu_25_M_30_ clusters, the HER becomes more favorable with a small value of |ΔG_H_| for Ag (0.10 eV), Cd (0.19 eV), Pd (0.92 eV) and Pt (0.76 eV) compared to the ΔG_PLS_ of the eCO_2_RR of Ag (0.37 eV), Cd (0.29 eV), Pd (1.61 eV) and Pt (1.23 eV). At this doping concentration, only Zn, with ΔG_PLS_ = 0.40 eV and ΔG_H_ = 0.49 eV, favors eCO_2_RR over HER. All core@shell models are more active towards HER than eCO_2_RR: the ΔG_H_ values of Ag (0.07 eV), Cd (0.45eV), Pd (−0.62 eV), Pt (−0.59 eV) and Zn(−0.27 eV) are lower than the ΔG_PLS_ values of Ag (0.76 eV), Cd (0.91 eV), Pd (1.12 eV), Pt (1.09 eV), and Zn (0.58 eV).

**Bimetallic clusters: CO_2_-to-HCOOH conversion.** For HCOOH formation, the steps are the CPET to convert adsorbed *CO_2_ to O-coordinated formate (*CO_2_ + H^+^ + e^−^ → *OCOH, ΔG_*OCOH_) and the CPET to convert adsorbed formate to liquid phase formic acid (*OCHO + H^+^ + e^−^ → HCOOH(l), Δ*G*_HCOOH_). In Figure 5, the ΔG_PLS_ value for HCOOH of Cu_54_M with CN6 for Ag (0.33 eV), Pd (0.33 eV) and Pt(0.37 eV) in the CuM cluster with CN8 for Ag(0.46 eV), Cd (0.46 eV), Pt(0.45 eV), Cu_43_M_12_ with Ag (0.39 eV), Pt (0.39 eV), Cu_25_M_30_ with Pd (0.19 eV), Pt (0.13 eV) and finally core@shell with Pd (0.33 eV), Pt (0.41 eV) are dominant over HER. We can explain this behaviour by considering the value of the d-band center (Table 2), which decreases for Ag, Cd and Zn with increasing doping concentration. The adsorption energy of the intermediates involved in the CO or HCOOH reaction pathway also decreases. Similarly, the higher position of the d-band center for Pd and Pt leads to an increase in the intermediate adsorption energy. Therefore, the core@shell model with low d-band center, Ag (–3.56 eV), Cd (–7.29 eV) and Zn (–6.23 eV), show poor catalytic performance, and Pd (–1.58 eV) and Pt (–1.92 eV) show good catalytic performance for HCOOH. Overall, the core@shell promotes the formation of HCOOH, and single metal-doped clusters show good catalytic performance for CO, except for Pt, which catalyzes HCOOH formation.

#### 3.3.2. Electrocatalytic CO_2_ conversion to CH_2_O, CH_3_OH, and CH_4_

**Figure 6:**
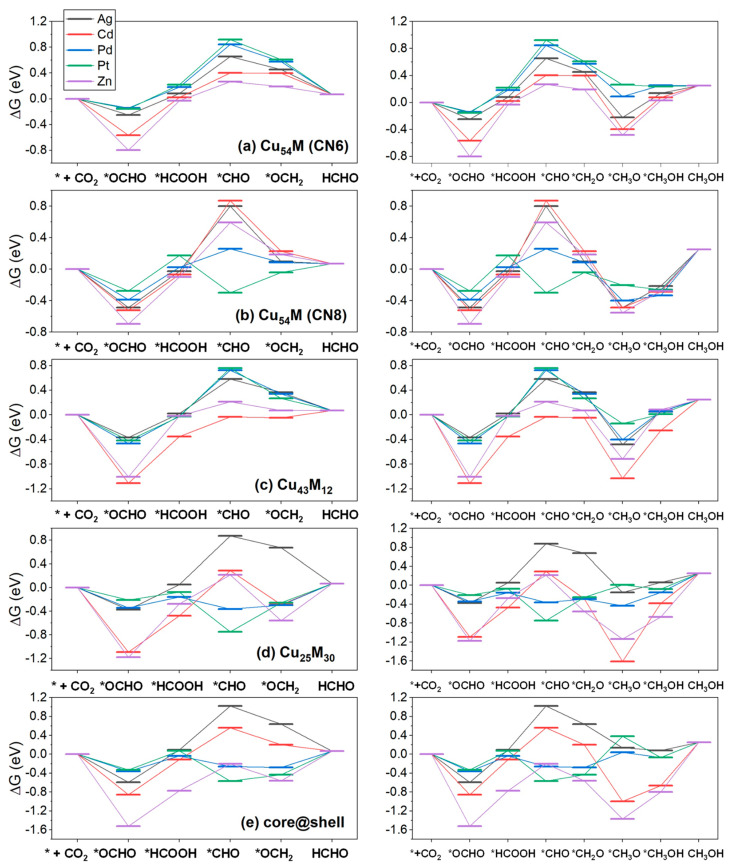
The Gibbs free energy diagram for the eCO_2_RR pathways to HCHO (left) and CH_3_OH (right) on the CuM clusters (M = Ag, Cd, Pd, Pt and Zn): (**a**) Cu_54_M with CN6, (**b**) Cu_54_M with CN8, (**c**) Cu_43_M_12_, (**d**) Cu_25_M_30_ and (**e**) core@shell.

The free energy diagrams for the eCO_2_RR to formaldehyde (CH_2_O), methanol (CH_3_OH) and methane (CH_4_) on the CuM clusters are reported in Figure 6.

**Bimetallic clusters: CH_2_O formation.** After the eCO_2_RR reduction to *CO or *HCOOH, further CPET steps generates three distinct intermediates: *CHO, *COH or *OCH. Among them, *CHO is the easiest to generate, as illustrated by the free energy diagram for the formation of these species, where the lowest ∆*G*_PLS_ is for COH. A subsequent CPET step leads to the formation of formaldehyde: *CHO + H^+^ + e^−^ → *OCH_2_ → * + CH_2_O(g). Due to the stronger O-affinity, CH_2_O prefers *OCHO than the *COOH route. CH_2_O generation shows lowest ΔG_PLS_ values on Cu_25_Pd_30_ (0.19 eV) and core@shell Cu@Pd (0.33 eV). As the ΔG_H_ values on Cu_25_Pd_30_ (0.92 eV) and core@shell Cu@Pd (0.62 eV) are higher than ΔG_PLS_ values, the CO_2_ conversion to CH_2_O is dominant over HER on these clusters. CuPd is also favorable towards CH_2_O formation as the Δ*G*_PLS_ values are higher than Δ*G*_H_ on these clusters. The values of ΔG_PLS_ are 0.45 eV for Cu_54_Pt (CN8), 0.49 eV for Cu_25_Pt_30_, and 0.44 eV for the Cu_54_Pt (CN8) and the core@shell Cu_25_Pt_30_ clusters. In comparison, the Δ*G*_H_ values on Cu_54_Pt (CN8), Cu_25_Pt_30_ and Cu@Pt are 0.46 eV, 0.76 eV and 0.59 eV, respectively. The Gibbs free energy diagram of the eCO_2_RR to CH_2_O on these systems is given as Figure 6a–e

**Bimetallic clusters: CH_3_OH formation.** The formation of CH_3_OH involves five CPET steps. The first three reduction steps coincide to the eCO_2_RR to CH_2_O. The *CHO is reduced to *CHOH or *OCH_2_ depending on if the O or C atoms ate protonated. This leads to four possible routes to convert CO_2_ to CH_3_OH:

*COOH → *CO → *CHO → *OCH_2_→ *OCH_3_ → *OHCH_3_;
*OCHO → *OCH_2_O → *OCH_2_OH → *O + CH_3_OH → *OH → * + H_2_O;
*OCHO → *HCOOH → *CHO → *CHOH → *CH_2_OH → *OHCH_3_;
*OCHO → *HCOOH → *CHO → *OCH_2_ → *OHCH_2_ → *OHCH_3_.

Out of these four paths, our calculations predict the last one as the most suitable for CH_3_OH formation. Similar to CH_2_O, the Cu_25_Pd_30_ and core@shell Cu@Pd show the lowest ΔG_PLS_, 0.28 eV and 0.33 eV, respectively, and still, these reactions are dominant over HER.

**Bimetallic clusters: CH_4_ formation.** The main 8-electron product of eCO_2_RR is CH_4_, involving eight CPET transfer steps. It follows five different reaction pathways:

*CHO → *CHOH → *CH → *CH_2_ → *CH_3_ → * + CH_4_;
*CHO → *CHOH → *CH_2_OH → *CH_2_→ *CH_3_ → * + CH_4_;
*CHO → *OCH_2_ → *OHCH_2_ → *OHCH_3_ → *OH + CH_4_ → * + H_2_O;
*CHO → *OCH_2_ → *OCH_3_ → *OHCH_3_ → *OH + CH_4_ → * + H_2_O;
*CHO → *OCH_2_ → *OCH_3_ → *O + CH_4_ → *OH → * + H_2_O.

The free energy diagrams along these pathways are given in Figures S9 and S10 of Supplementary Information.

**Figure 7:**
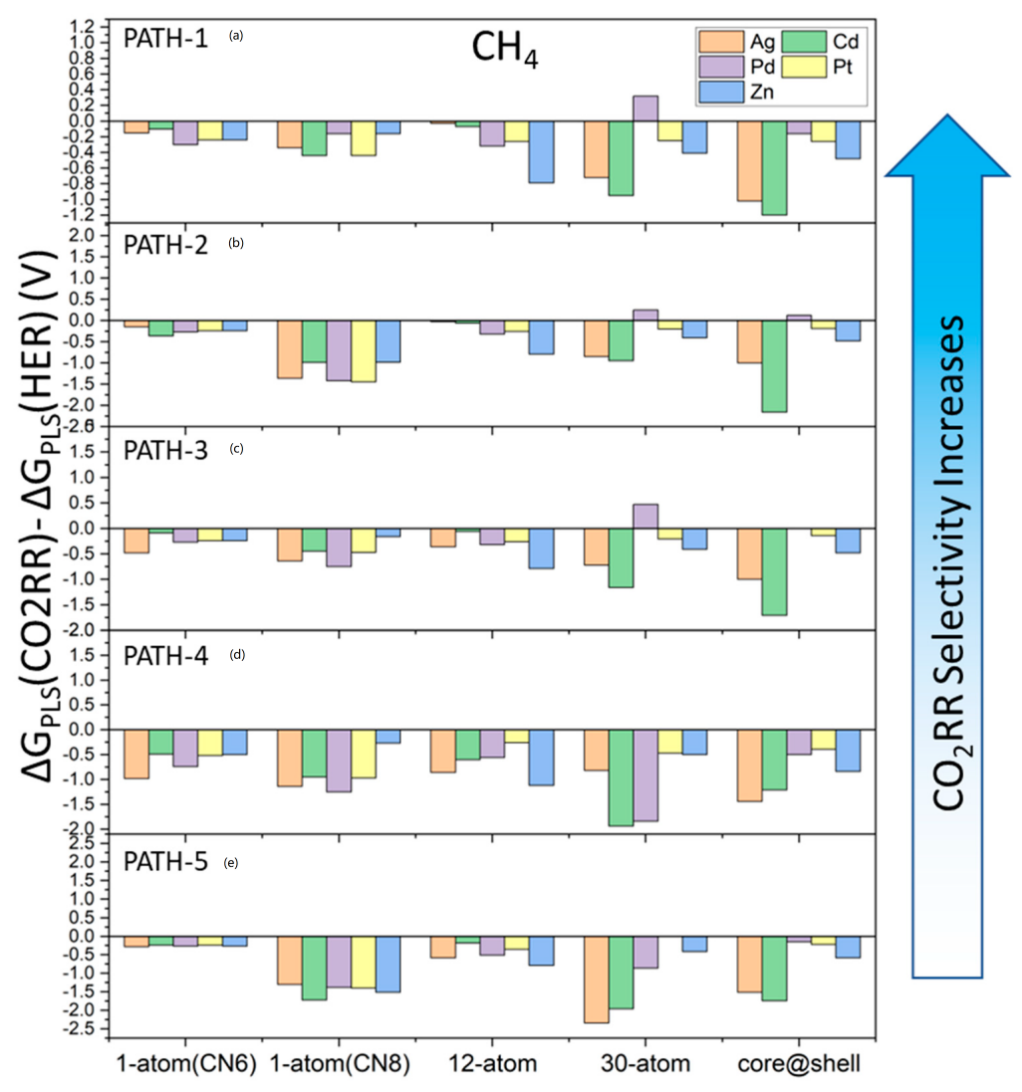
The limiting potential difference (∆∆*G*_PLS_) between CO_2_RR to CH_4_ and HER on the bimetallic CuM clusters (M = Ag, Cd, Pd, Pt and Zn): (**a**) Cu_54_M with CN6, (**b**) Cu_54_M with CN8, (**c**) Cu_43_M_12_, (**d**) Cu_25_M_30_ and (**e**) core@shell.

**Competition between CH_4_ and H_2_ formation.** HER is a competitive reaction in eCO_2_RR and can reduce the efficiency of the eCO_2_RR reaction leading to poor selectivity of the catalyst. To evaluate the selectivity of eCO_2_RR vs. HER, we have reported in Figure 7 the limiting potential difference ∆∆G_PLS_ = ∆G_PLS_(eCO_2_RR) − ∆G_H_(HER) for the five reaction pathways leading to the formation of CH_4_ on the CuM clusters. The higher the (positive) value of ∆∆*G*_PLS_, the higher the selectivity for CH_4_. Only CuPd shows good catalytic performance towards CH_4_ formation. Through the reaction pathway (1), Cu_25_Pd_30_ has a positive ∆∆*G*_PLS_. The Cu_43_Ag_12_ and Cu_43_Cd_12_ clusters show a small negative ∆∆G_PLS_ value. For pathways (2) and (3), the Cu_25_Pd_30_ and Cu@Pd show a positive ∆∆G_PLS_ value and hence these two systems are selective towards CH_4_. Like pathway (1), the Cu_43_Ag_12_ and Cu_43_Cd_12_ show a very small ∆∆*G*_PLS_ for (2) which makes them also good candidates for catalyzing CH_4_ formation. Finally, Cu_13_Cd_42_ shows a small ∆∆*G*_PLS_, which can be explained based on the d-band center and coordination environment: at the same doping concentration (1-atom) with CN6 and CN8, the 1-atom at CN6 show significantly low overpotential for all pathways leading to CH_4_ generation. Single-doped clusters with CN6 and CN8 have similar d-band center values (Table 2), only the coordination environment is different, which means that the CN environment has a significant impact on the catalytic performance. Furthermore, the d-band center value increases with an increase in doping concentration for Cu-Pd and Cu-Pt catalysts. Consequently, there is strong adsorption of the intermediates involved in the reaction (pathways 1 to 5). However, with M = Ag, Cd and Zn, the values of d-band centers decreases, which leads to weak adsorption of the intermediates and poor catalytic performance towards CH_4_ formation.

#### 3.3.3. Selectivity

**Figure 8:**
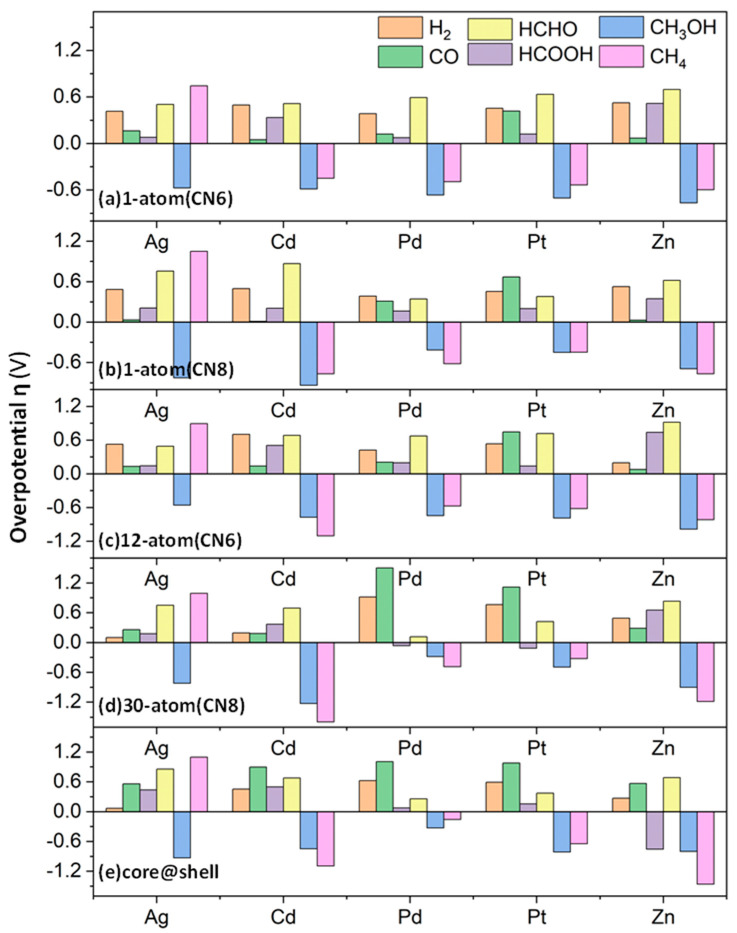
The overpotentials (η) for the electrocatalytic formation of H_2_, CO, HCOOH, CH_2_O, CH_3_OH and CH_4_ on the CuM clusters (M = Ag, Cd, Pd, Pt and Zn): (**a**) Cu_54_M with CN6, (**b**) Cu_54_M with CN8, (**c**) Cu_43_M_12_, (**d**) Cu_25_M_30_ and (**e**) core@shell.

The overpotentials (η) to C1 products for all CuM systems, summarized in Figure 8**,** were computed from the equilibrium (*U*_eq_) and limiting (*U*_L_) potentials (Figure 5 and Figure 6). The values of *U*_eq_ for CO, HCOOH, CH_2_O, CH_3_OH and CH_4_ are 0.12 V, 0.25 V, 0.07 V, 0.02 V and 0.17 V, respectively. For the Cu clusters doped with Ag, Cd and Zn, an increase in metal doping, particularly after 30-atom, leads to the HER becoming dominant over other C1 products. This behaviour is clearly noticeable for the single atom doped clusters, Cu_54_M with M = Ag, Cd and Zn, which shows higher η values for HER than the corresponding core@shell systems. The values of the overpotential also show that a single atom doped system supports either CO or HCOOH. Therefore, small doping does not promote CH_3_OH or CH_4_ formation. As metal doping increases, the Cu-Pd and Cu-Pt clusters show lower overpotential for CH_2_O, CH_3_OH and CH_4_. Only Cu_25_Pt_30_ and Cu_13_@Pd_42_ have the lowest overpotential for CH_4_. 

## 4. Conclusions

In this work, the catalytic properties towards the electrochemical CO_2_ reduction reaction of a series of icosahedral 55-atom Cu-based clusters doped with Ag, Cd, Pd, Pt and Zn were investigated using density functional theory calculations. The adsorption and activation of CO_2_ on these clusters and all possible reaction paths that lead to the CO_2_ reduction to C1 products (CO, HCOOH, CH_2_O, CH_3_OH and CH_4_) were considered. Apart from the composition effects, the role of the coordination environment of the metal dopant on the catalytic performance of copper-based clusters was also investigated, with the results showing that nanoclusters with eight-coordinated metal dopants have better catalytic activity towards CO_2_ activation. Single-atom doping with Cd and Zn is the best candidate for the CO_2_-to-CO conversion, while core@shell with Ag, Pd and Pt is a good choice for formic acid or formaldehyde formation. The CuPt and CuPd systems show the lowest overpotential for methane formation. This work identifies the influence of size, metal coupling and metal coordination on CO_2_ activation and intermediate stability and, consequently, the structure-property relationship in Cu-based mono and bi-metallic clusters for the selective CO_2_ conversion to value-added C1 products.

## Figures and Tables

**Table 1:** The bond lengths (Å), formation energy (eV), HOMO-LUMO gap (Δ_H-L_, eV), Bader charge difference (∆Q_M_, Coulomb), and surface energy (*γ*, eV) of the Cu and CuM (M = Ag, Cd, Pd, Pt and Zn) nanoclusters.

	Bond Length		Formation Energy	Δ_H−L_	∆Q_M_	*γ*
Pristine Cu_55_ Nanocluster						
**Cu_55_**	2.51		−2.99	―	―	−0.56
**1-atom doping on CN6**						
**Cu_54_Ag_1_**	2.69		−3.51	0.0001	−0.12	−0.56
**Cu_54_Cd_1_**	2.73		−3.48	0.0412	0.16	−0.54
**Cu_54_Pd_1_**	2.59		−3.53	0.1013	−0.37	−0.58
**Cu_54_Pt_1_**	2.56		−3.56	0.0441	−0.64	−0.59
**Cu_54_Zn_1_**	2.54		−3.48	0.0005	0.13	−0.58
**1-atom doping on CN8**						
**Cu_54_Ag_1_**	2.69		−3.52	0.0488	−0.08	−0.56
**Cu_54_Cd_1_**	2.73		−3.49	0.0349	0.14	−0.54
**Cu_54_Pd_1_**	2.59		−3.54	0.0431	−0.30	−0.58
**Cu_54_Pt_1_**	2.56		−3.57	0.0481	−0.63	−0.59
**Cu_54_Zn_1_**	2.54		−3.49	0.0414	0.17	−0.58
**12-atom doping on CN6**						
**Cu_43_Ag_12_**	2.67		−3.30	0.0858	−0.12	−0.51
**Cu_43_Cd_12_**	2.75		−2.90	0.0104	0.14	−0.42
**Cu_43_Pd_12_**	2.59		−3.59	0.0682	−0.32	−0.64
**Cu_43_Pt_12_**	2.56		−3.96	0.0824	−0.58	−0.68
**Cu_43_Zn_12_**	2.54		−2.98	0.1721	0.09	−0.49
**30-atom doping on CN8**						
	**Cu-M**	**M-M**				
**Cu_25_Ag_30_**	2.65	2.81	−2.93	0.0305	−0.06	−0.46
**Cu_25_Cd_30_**	2.67	3.01	−1.95	0.0016	0.08	−0.26
**Cu_25_Pd_30_**	2.59	2.70	−3.49	0.0568	−0.15	−0.71
**Cu_25_Pt_30_**	2.59	2.69	−4.45	0.0524	−0.26	−0.82
**Cu_25_Zn_30_**	2.53	2.75	−2.12	0.0980	0.09	−0.34
**Core@shell**						
**Cu_13_Ag_42_**	2.81		−2.81	0.0835	−0.14	−0.40
**Cu_13_Cd_42_**	2.95		−1.31	0.0131	−0.93	−0.15
**Cu_13_Pd_42_**	2.68		−3.40	0.0168	0.78	−0.72
**Cu_13_Pt_42_**	2.62		−4.72	0.0527	0.69	−0.86
**Cu_13_Zn_42_**	2.56		−1.53	0.0173	−0.93	−0.23

**Table 2:** The adsorption energies (*E*_ads_, eV), bond angles (*θ*_OCO_, °), charge difference (∆Q_M_, Coulomb) bond elongations (∆l_CO_, Å) and d-bands center (*δ*_d_, eV) of CO_2_ adsorbed on the Cu-M nanoclusters (M = Ag, Cd, Pd, Pt and Zn). The symbols η(Cu, C) and η(M, C) refer to the coordination of the C atom of the adsorbed CO_2_ molecule to the Cu and M atoms, respectively.

	*E*_ads_		*θ*(O-C-O)		∆Q_M_		∆l_CO_	*δ*_d_
	η(Cu,C)	η(M,C)	η(Cu,C)	η(M,C)	η(Cu,C)	η(M,C)		
Cu_55_	−0.01	―	33.40	―	―	―	0.05	−2.27
**1-atom doping (CN6)**								
Cu_54_Ag_1_	−0.20	−0.20	0.340	0.460	0.04	0.04	0.00	−2.28
Cu_54_Cd_1_	0.07	−0.18	43.84	0.250	0.61	0.04	0.09	−2.38
Cu_54_Pd_1_	−0.20	−0.33	0.710	40.12	0.04	0.51	0.17	−2.29
Cu_54_Pt_1_	−0.19	−0.56	0.750	44.67	0.04	0.57	0.21	−2.29
Cu_54_Zn_1_	−0.13	−0.20	47.45	0.300	0.70	0.04	0.23	−2.36
**1-atom doping (CN8)**								
Cu_54_Ag_1_	−0.18	−0.19	0.65	0.42	0.04	0.04	0.00	−2.28
Cu_54_Cd_1_	0.14	−0.17	41.3	0.41	0.58	0.05	0.15	−2.34
Cu_54_Pd_1_	−0.18	−0.22	0.72	45.52	0.05	0.63	0.17	−2.28
Cu_54_Pt_1_	−0.21	−0.43	1.28	50.97	0.63	0.71	0.24	−2.26
Cu_54_Zn_1_	0.05	0.26	48.8	52.21	0.76	0.86	0.27	−2.34
**12-atom doping (CN6)**								
Cu_43_Ag_12_	−0.21	−0.21	0.660	0.300	0.04	0.04	0.01	−2.67
Cu_43_Cd_12_	−0.44	−0.23	48.50	45.60	0.75	0.68	0.23	−4.03
Cu_43_Pd_12_	−0.22	−0.25	0.470	38.02	0.04	0.49	0.19	−2.12
Cu_43_Pt_12_	−0.07	−0.34	1.260	42.38	0.78	0.53	0.29	−2.05
Cu_43_Zn_12_	−0.22	0.13	49.22	49.30	0.75	0.77	0.27	−3.72
**30-atom doping (CN8)**								
Cu_25_Ag_30_	−0.22	−0.23	0.490	0.620	0.04	0.04	0.00	−3.12
Cu_25_Cd_30_	−0.09	−0.64	32.19	0.240	0.65	0.05	0.27	−5.27
Cu_25_Pd_30_	0.00	−0.52	35.30	42.33	0.38	0.48	0.25	−1.49
Cu_25_Pt_30_	−0.34	−0.56	1.070	44.72	0.02	0.49	0.29	−1.75
Cu_25_Zn_30_	−0.50	−0.37	49.68	50.85	0.75	0.82	0.23	−4.61
**42-atom doping (core@shell)**								
Cu_13_Ag_42_	−0.15	−0.21	0.37	0.34	0.03	0.04	0.00	−3.56
Cu_13_Cd_42_	−0.11	−0.20	0.75	0.46	0.04	0.06	0.00	−7.29
Cu_13_Pd_42_	−0.13	−0.22	1.96	2.04	0.04	0.04	0.00	−1.58
Cu_13_Pt_42_	−0.10	−0.17	0.82	1.09	0.02	0.03	0.00	−1.92
Cu_13_Zn_42_	−0.20	−0.18	0.10	0.41	0.02	0.04	0.00	−6.23

## Data Availability

The data presented in this study are available on request from the corresponding author.

## Supplementary Materials

The following supporting information can be downloaded at: https://www.mdpi.com/article/10.3390/nano13010087/s1, Table S1: The energies (E), zero-point energies (ZPE), and entropies (S) of H_2_(g), CO_2_(g) and CO(g), and H_2_O; Table S2: The atomic, covalent and Van der Waals radii, the electronegativity difference, electronic configuration, and calculated value of segregation energies (in eV) [57,58]; Figure S1: (a) The binding energy and (b) HOMO-LUMO (H-L) gap of Cu-M clusters with increasing doping concentration; Figure S2: The structure and adsorption energies (in eV) of COOH adsorbed on the Cu-M clusters; Figure S3: The structure and adsorption energies (in eV) of CO adsorbed on the Cu54M clusters with CN6 and CN8 nano-catalysts; Figure S4: The structure and adsorption energies (in eV) of CO on the Cu_43_M_12_ and Cu_25_M_30_ clusters; Figure S5: The structure and adsorption energies (in eV) of CO on the Cu_43_M_12_ and Cu_25_M_30_ clusters; Figure S6: The structure and adsorption energies of H adsorbed on the Cu_43_M_12_ and Cu_25_M_30_ clusters at the Top, Hollow and Bridge positions; Figure S7: The structure and adsorption energies of H adsorbed on the Cu_43_M_12_ and Cu_25_M_30_ clusters at Top, Hollow and Bridge positions; Figure S8: The structures and adsorption energies of H adsorbed on the core@shell clusters at Top, Hollow and Bridge positions; Figure S9. Gibbs free energy diagram for the CH_4_ formation on CuM clusters along pathways 1 to 5; Figure S10. Gibbs free energy diagram for CHO (a) and COH (b) formation on CuM clusters.
