# Supplementary material for: Optimal Icosahedral Copper-Based Bimetallic Clusters for the Selective Electrocatalytic CO2 Conversion to One Carbon Products
Source: Nanomaterials (Basel). 2022 Dec 24;13(1):87. doi: 10.3390/nano13010087 (PMC9823659; doi:10.3390/nano13010087)
Supplement: Supplementary file 1 [file nanomaterials-13-00087-s001.zip › nanomaterials-2068546-supplementary.pdf]

Supplementary Information

Table S1. The energies (E), zero-point energies (ZPE), and entropies (S) of H_2(g), CO_2(g) and CO(g), and H_2O. The entropies of H_2(g), CO_2(g) and CO(g) were calculated at 1 atm. The entropy of H_2O (g=l) was calculated at 0.035 atm, which corresponds to the vapor pressure of liquid water.

Gas Phase	E	ZPE	C_pdT	TS	ΔG
**H_2**	-6.71	0.27	0.09	-0.41	-6.76
**H_2O**	-14.2	0.56	0.1	-0.65	-14.19
**CO(g)**	-14.43	0.13	0.09	-0.59	-14.8
**CO_2**	-22.96	0.27	0.1	-0.65	-23.24

Table S2. The atomic, covalent and Van der Waals radii, the electronegativity difference, electronic configuration, and calculated value of segregation energies (in eV).

Atomic Symbol	Radius [Å][1][2]					Segregation Energy (eV)	
	**Atomic**	**Covalent**	**Van-der-Waals**	**ΔE_N (eV)**	**Electronic Configuration**	**CN6**	**CN8**
**Cu**	1.45	1.38	1.40	0.00	$4s^1 3d^{10}$		
**Ag**	1.65	1.53	1.72	0.03	$5s^1 4d^{10}$	-1.46	-1.53
**Cd**	1.61	1.48	1.58	0.21	$5s^2 4d^{10}$	-2.50	-2.60
**Pd**	1.69	1.31	1.63	0.30	$4p^6 4d^{10}$	-0.46	-0.59
**Pt**	1.77	1.28	1.75	0.38	$5d^9 6s^1$	-0.20	-0.38
**Zn**	1.42	1.31	1.39	0.25	$4s^2 3d^{10}$	-0.57	-0.68

[1] Alvarez, S. A cartography of the van der Waals territories. *Dalt. Trans.* **2013**, *42,* 8617–8636. doi: 10.1039/c3dt50599e.

[2] Cordero,B.; Gómez, V.; Platero-Prats, A.E.; Revés, M.; Echeverría, J.; Cremades, E.; Barragán, F.; Alvarez, S. Covalent radii revisited. *J. Chem. Soc. Dalt. Trans.* **2008**, *21,* 2832–2838. doi: 10.1039/b801115j.

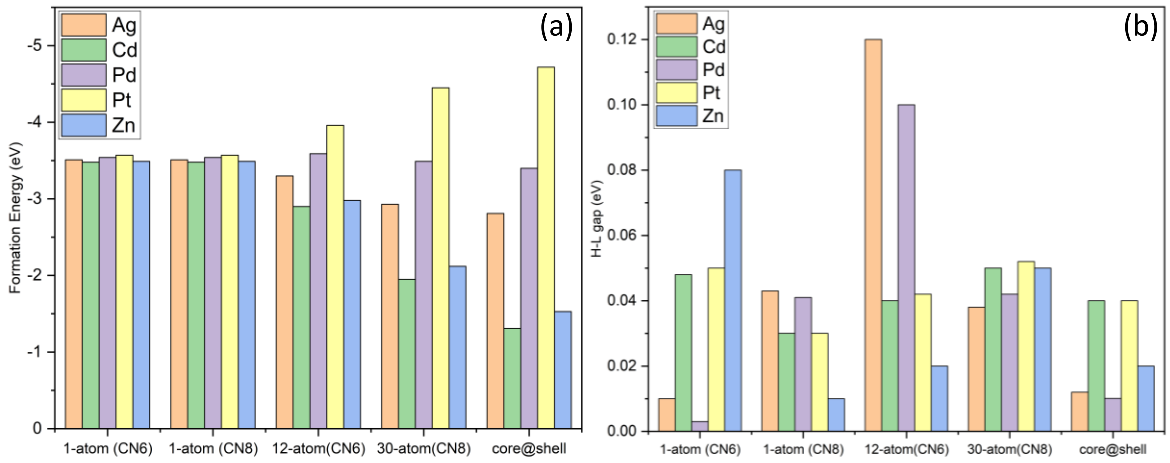

Figure S1. (a) The binding energy and **(b)** HOMO-LUMO (H-L) gap of Cu-M clusters with increasing doping concentration.

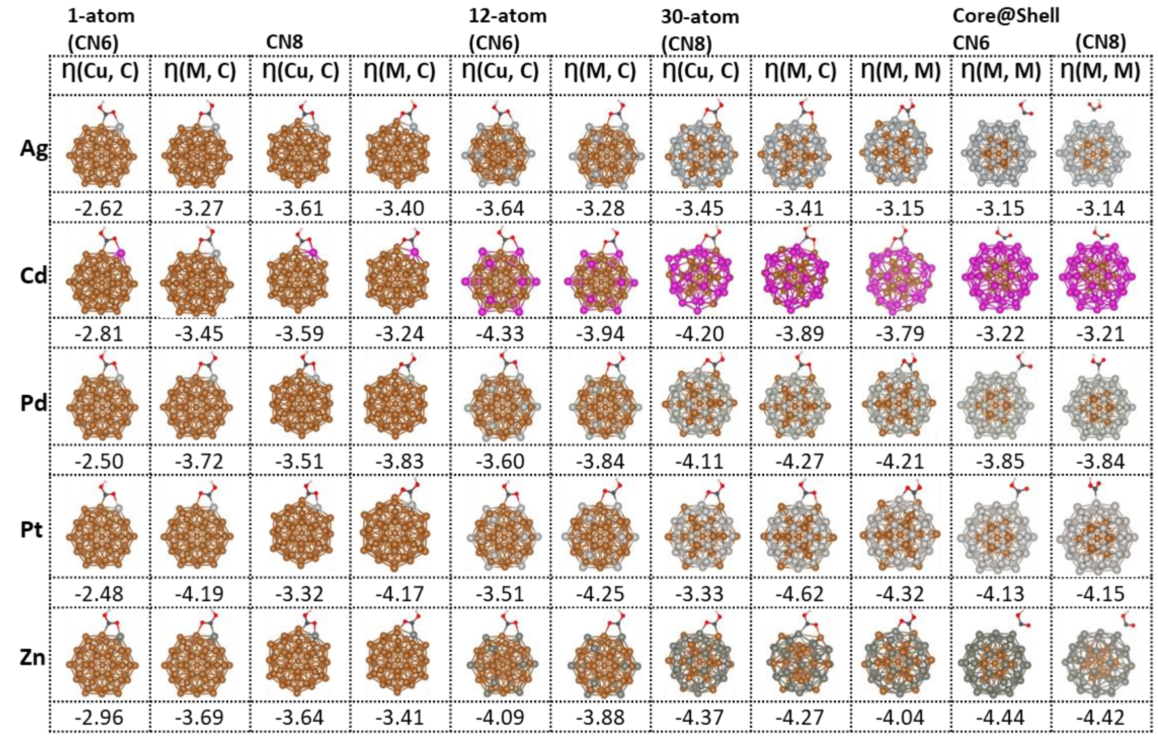

Figure S2. The structure and adsorption energies (in eV) of COOH adsorbed on the Cu-M clusters.

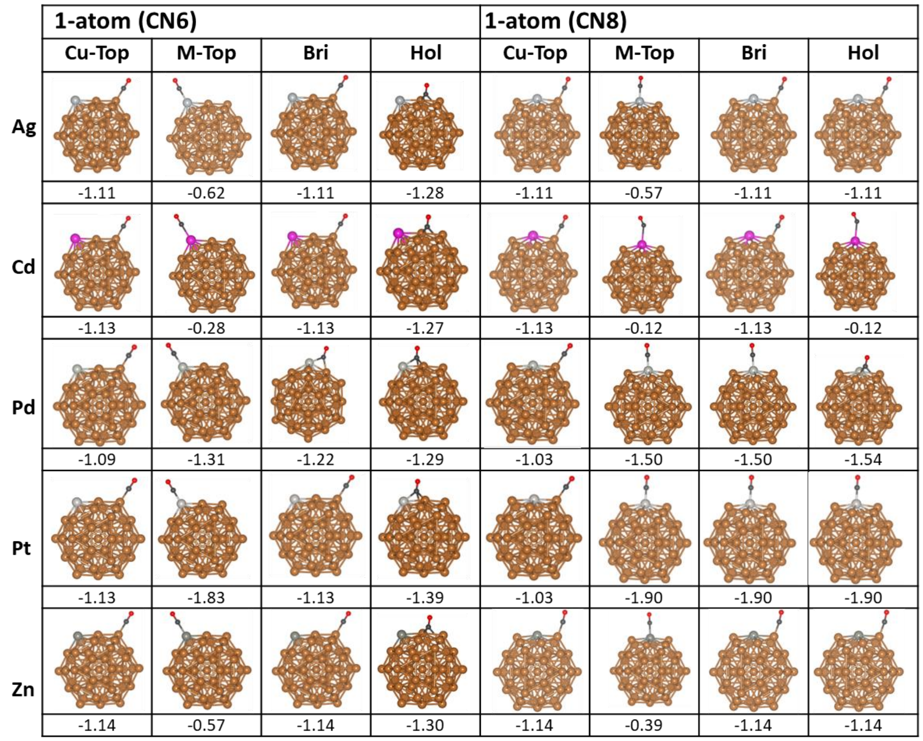

Figure S3. The structure and adsorption energies (in eV) of CO adsorbed on the $Cu_{54}M$ clusters with CN6 and CN8 nano-catalysts.

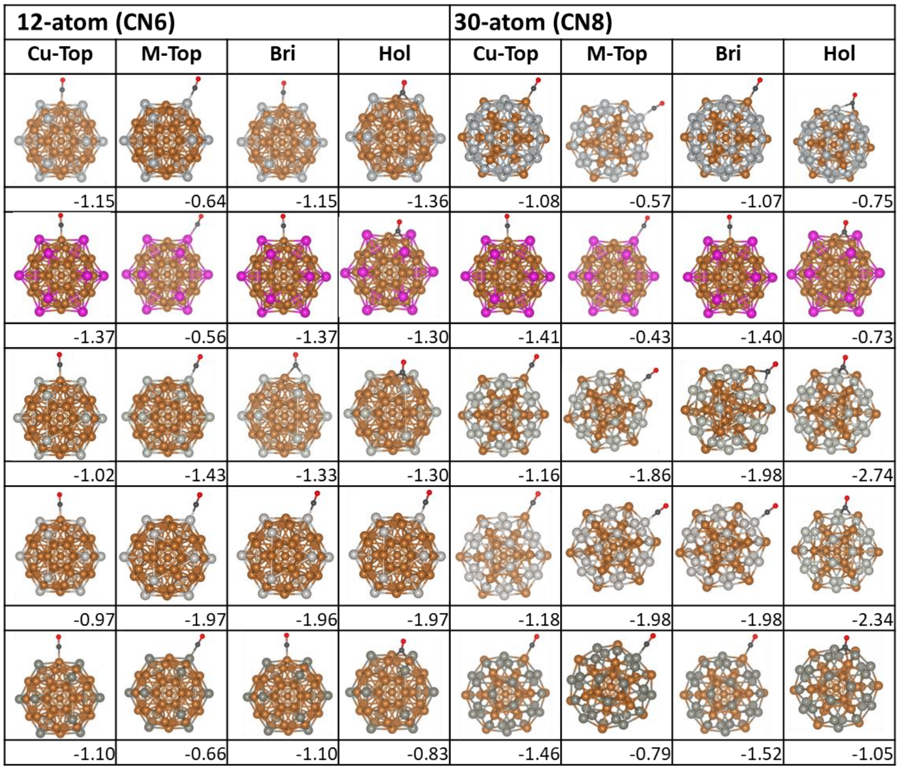

Figure S4. The structure and adsorption energies (in eV) of CO on the $Cu_{43}M_{12}$ and $Cu_{25}M_{30}$ clusters.

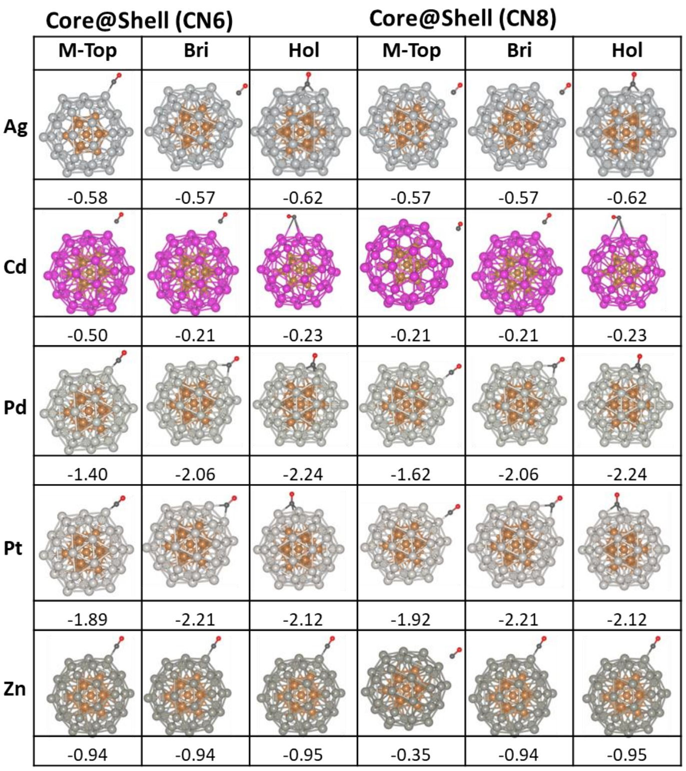

Figure S5. The structure and adsorption energies (in eV) of CO on the $Cu_{43}M_{12}$ and $Cu_{25}M_{30}$ clusters.

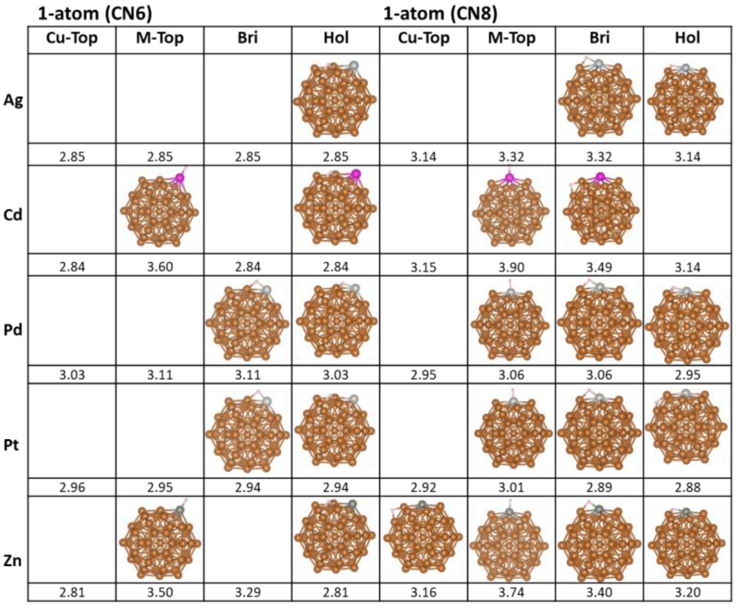

Figure S6. The structure and adsorption energies of H adsorbed on the $Cu_{43}M_{12}$ and $Cu_{25}M_{30}$ clusters at the Top, Hollow and Bridge positions.

	12-atom (CN6)				30-atom (CN8)			
	Cu-Top	M-Top	Bri	Hol	Cu-Top	M-Top	Bri	Hol
Ag			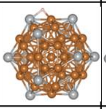	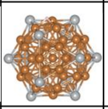			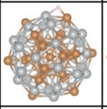	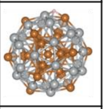
	3.35	2.81	3.35	2.82	3.24	3.24	3.32	3.24
Cd		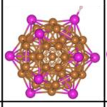	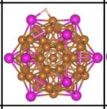	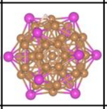		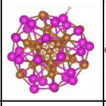	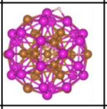	
	2.92	3.12	2.92	2.63	3.54	3.46	3.49	3.46
Pd			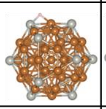	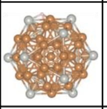				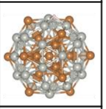
	2.96	2.91	2.96	2.91	3.54	3.46	3.49	3.46
Pt			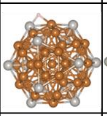	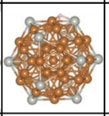		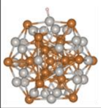	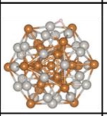	
	2.80	2.80	2.80	2.87	2.60	2.71	2.57	2.60
Zn		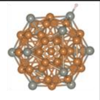	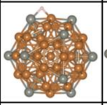	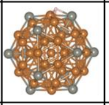		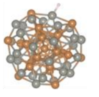	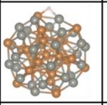	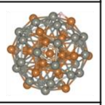
	3.20	3.35	3.20	3.14	2.85	3.15	2.87	2.93

Figure S7. The structure and adsorption energies of H adsorbed on the $Cu_{43}M_{12}$ and $Cu_{25}M_{30}$ clusters at Top, Hollow and Bridge positions.

	Core@Shell (CN6)			Core@Shell (CN8)		
	M-Top	Bri	Hol	M-Top	Bri	Hol
Ag		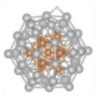	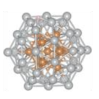		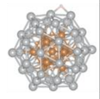	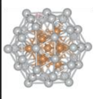
	3.52	3.52	3.40	3.53	3.51	3.41
Cd	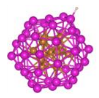	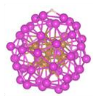			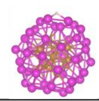	
	4.10	4.10	4.10	4.10	4.10	4.10
Pd		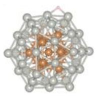	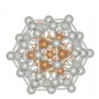		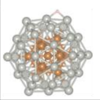	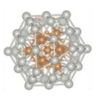
	2.88	2.88	2.72	2. 85	2.82	2.73
Pt		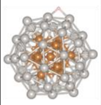	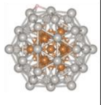	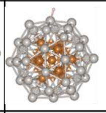	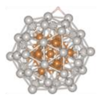	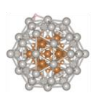
	2.74	2.74	2.81	2.74	2.75	2.82
Zn	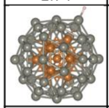			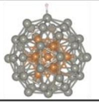	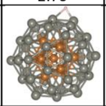	
	2.69	2.69	2.68	2.68	2.67	2.69

Figure S8. The structures and adsorption energies of H adsorbed on the core@shell clusters at Top, Hollow and Bridge positions.

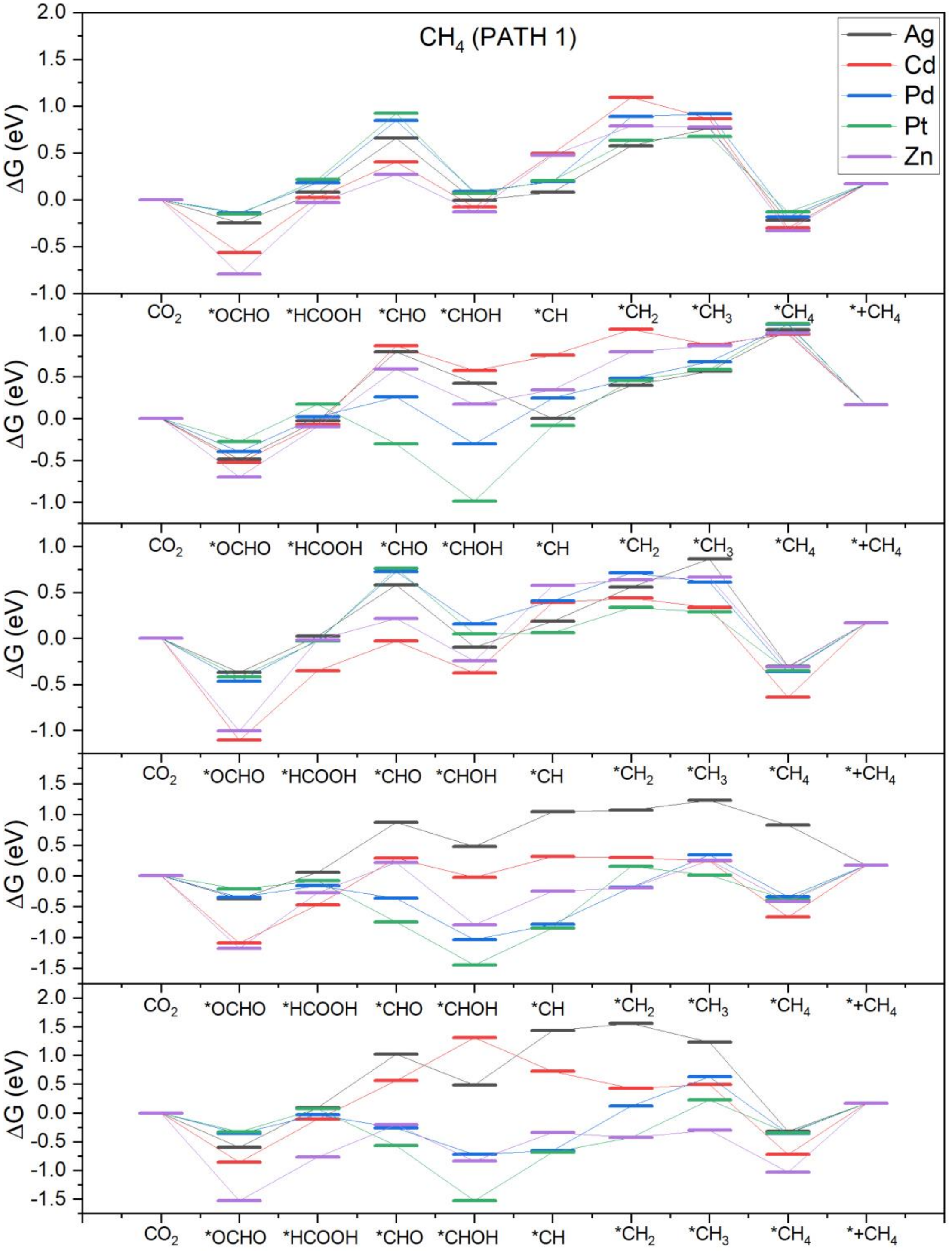
CH4 (PATH 1)
Ag
Cd
Pd
Pt
Zn
ΔG (eV)
CO2
*OCHO
*HCOOH
*CHO
*CHOH
*CH
*CH2
*CH3
*CH4
*+CH4

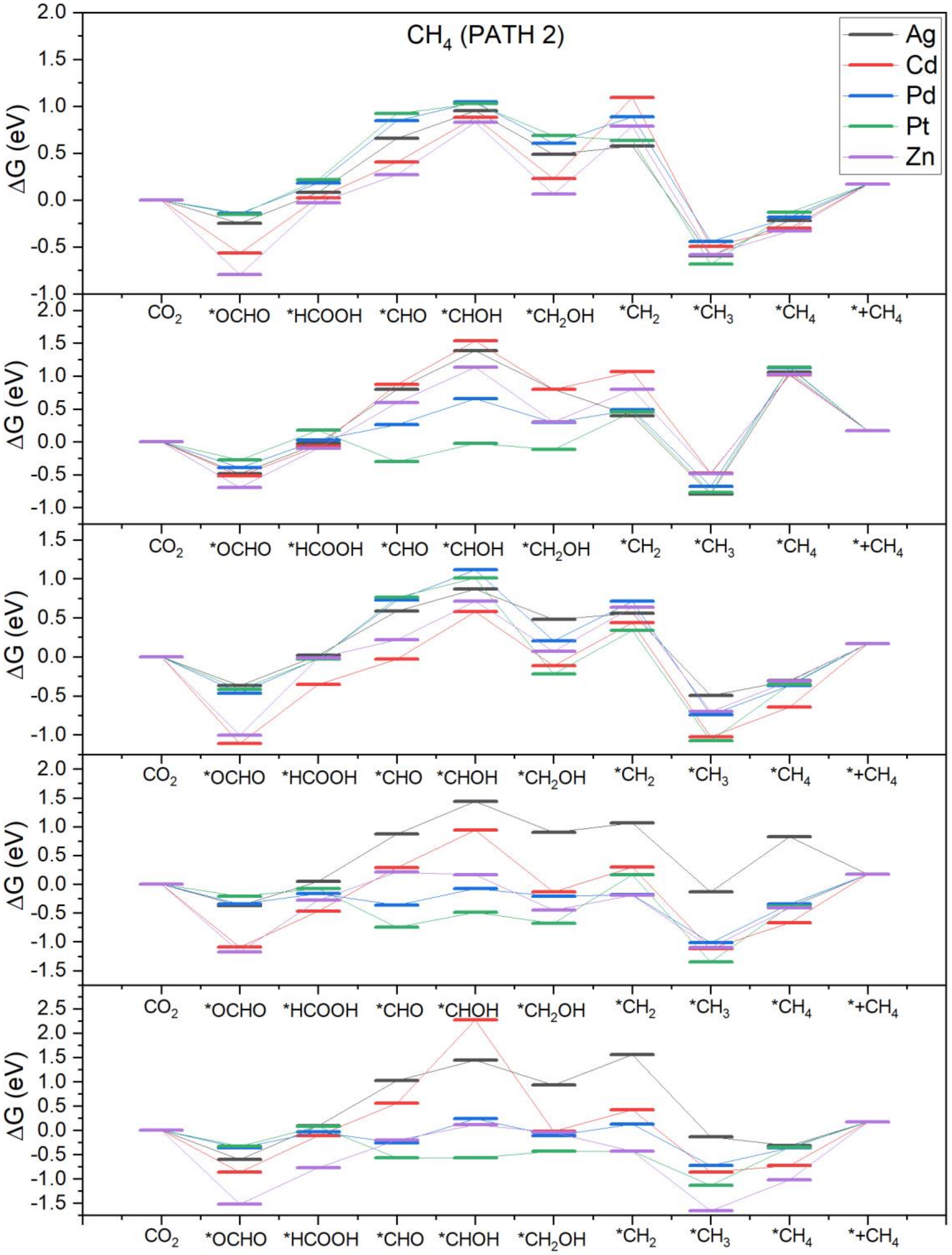

CH$_4$ (PATH 2)
Ag
Cd
Pd
Pt
Zn
ΔG (eV)
CO$_2$
*OCHO
*HCOOH
*CHO
*CHOH
*CH$_2$OH
*CH$_2$
*CH$_3$
*CH$_4$
*+CH$_4$

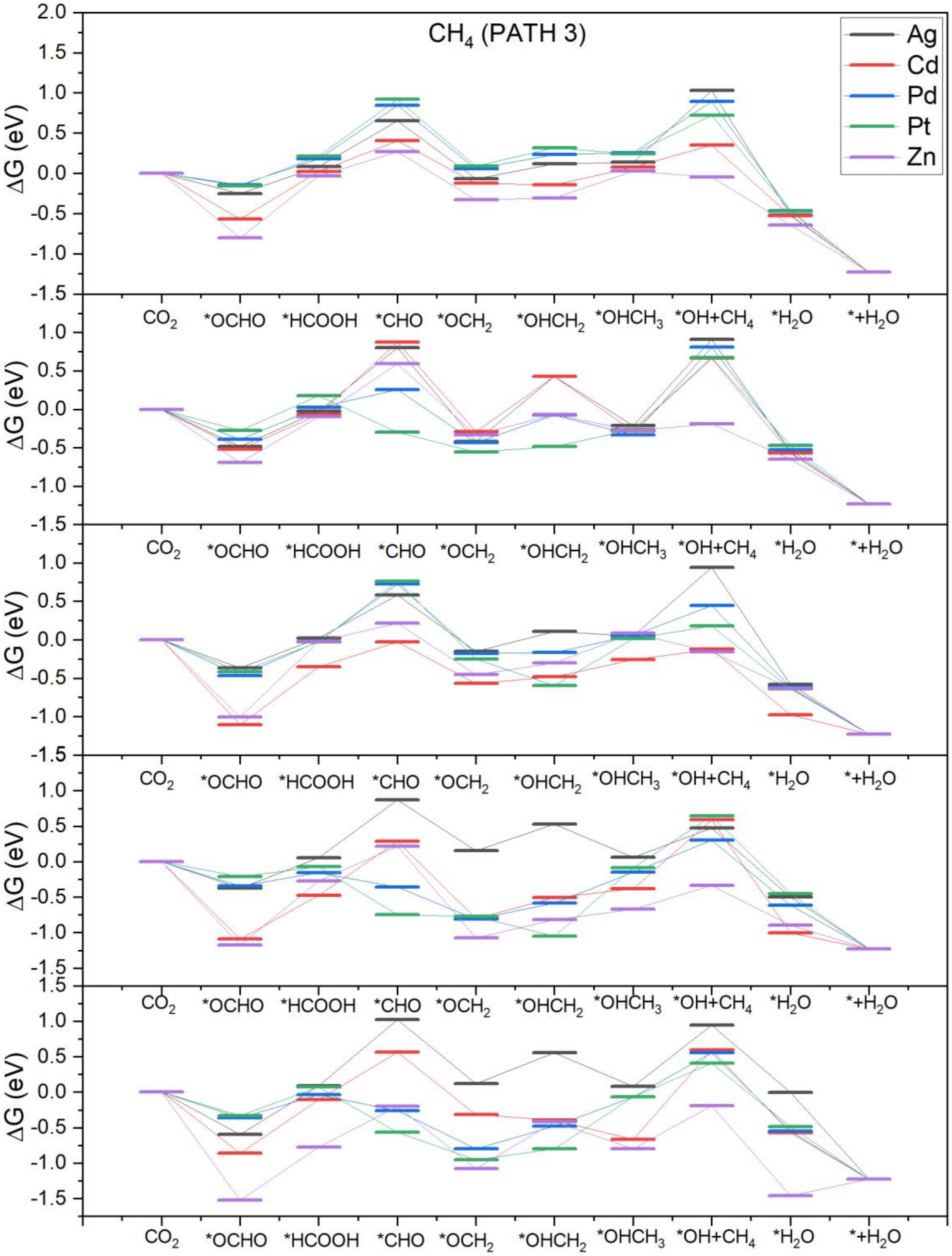

CH_4 (PATH 3)
Ag
Cd
Pd
Pt
Zn
ΔG (eV)
CO_2
*OCHO
*HCOOH
*CHO
$*OCH_2$
$*OHCH_2$
$*OHCH_3$
$*OH+CH_4$
$*H_2O$
$*+H_2O$

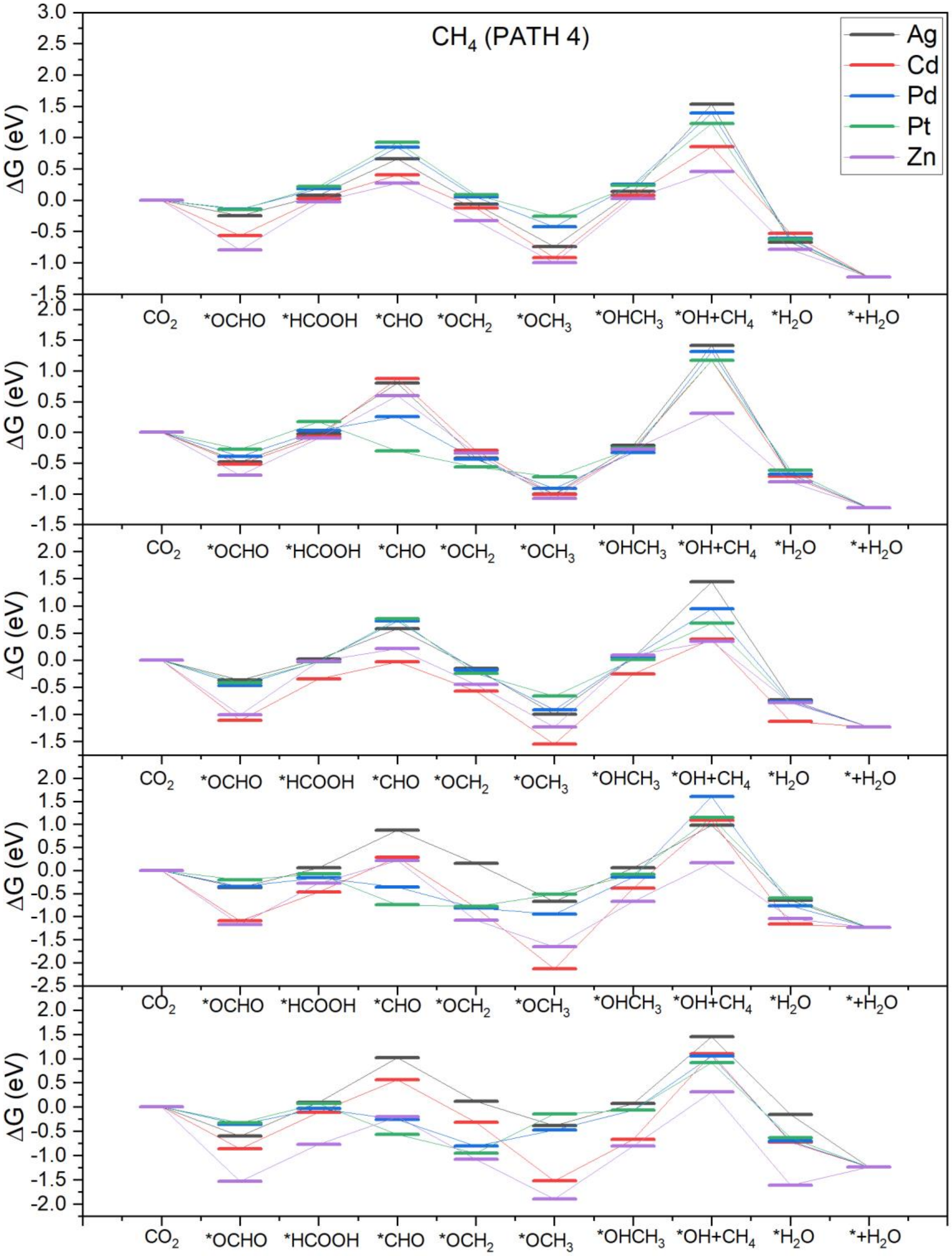
CH$_4$ (PATH 4)
Ag
Cd
Pd
Pt
Zn
ΔG (eV)
CO$_2$
*OCHO
*HCOOH
*CHO
*OCH$_2$
*OCH$_3$
*OHCH$_3$
*OH+CH$_4$
*H$_2$O
*+H$_2$O

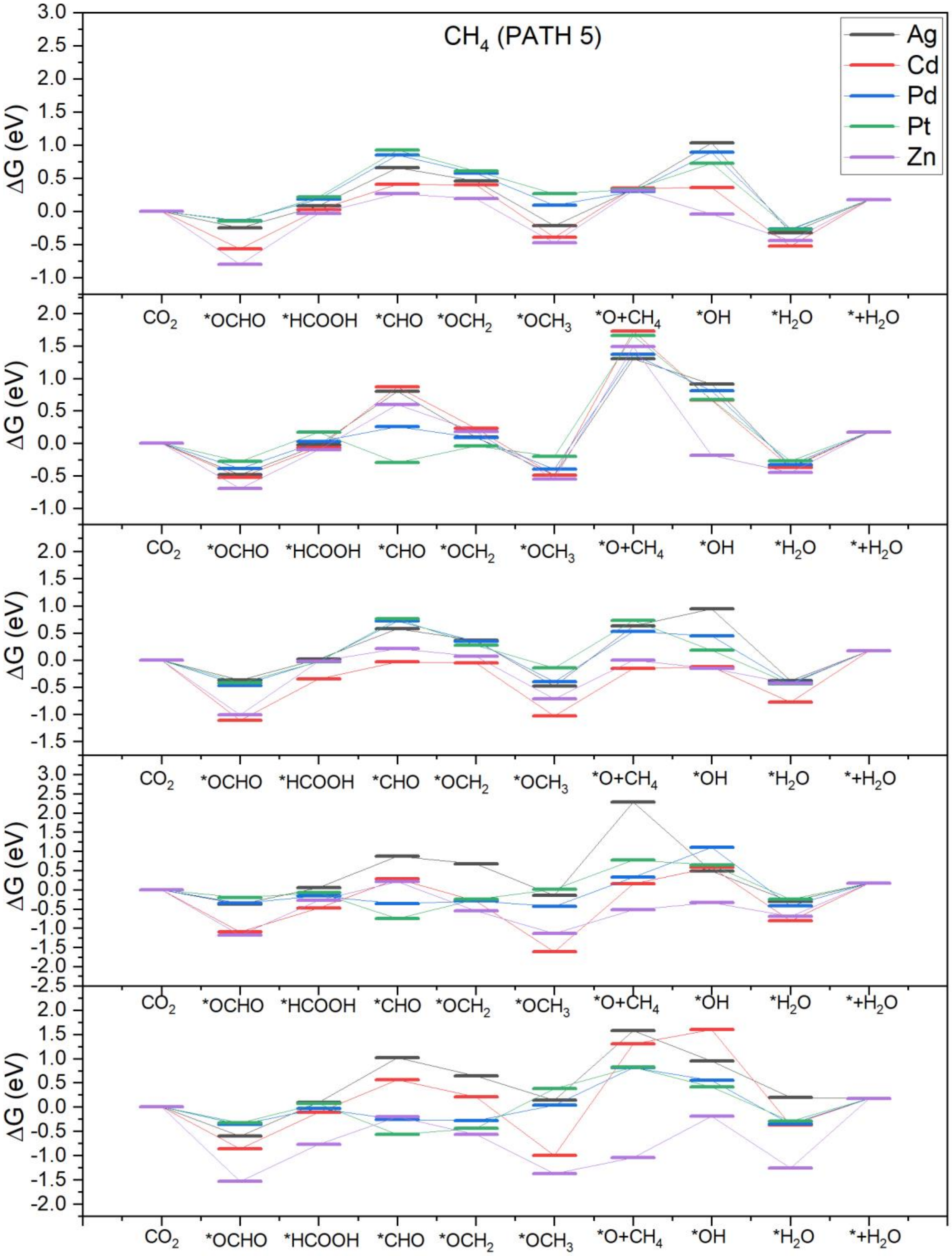

Figure S9. Gibbs free energy diagram for the CH_4 formation on CuM clusters along pathways 1 to 5: **(1)** ∗CHO → ∗CHOH → ∗CH → ∗CH_2 → ∗CH_3 → ∗ + CH_4; **(2)** ∗CHO → ∗CHOH → ∗CH_2OH → ∗CH_2→ ∗CH_3 → ∗ + CH_4; **(3)** ∗CHO → ∗OCH_2 → ∗$OHCH_2$ → ∗$OHCH_3$ → ∗OH + CH_4 → * + H_2O; **(4)** ∗CHO → ∗OCH_2 → ∗OCH_3 → ∗$OHCH_3$ → ∗OH + CH_4 → ∗ + H_2O; **(5)** ∗CHO → ∗OCH_2 → ∗OCH_3 → ∗O + CH_4 → ∗OH → ∗ + H_2O.

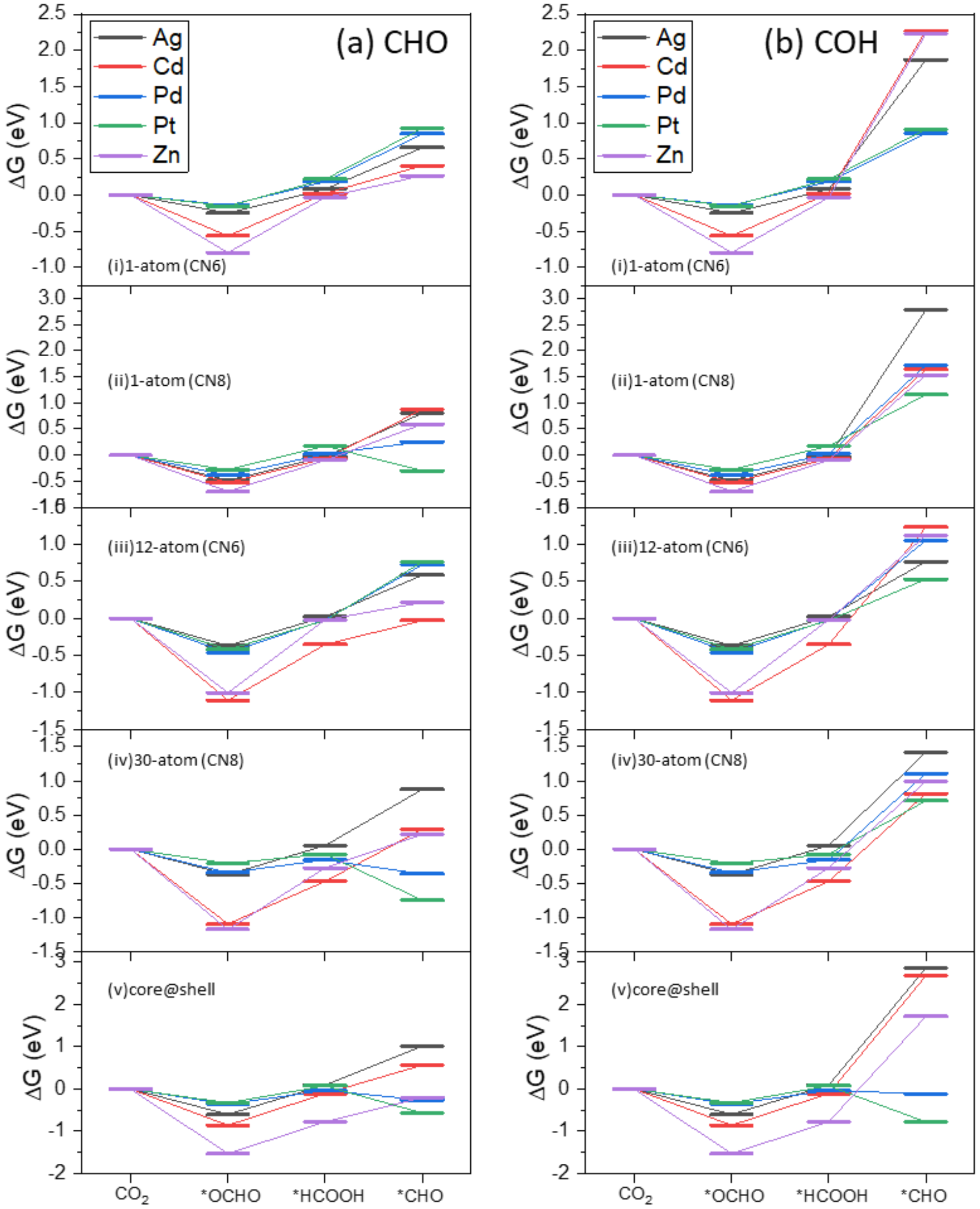

Figure S10. Gibbs free energy diagram for CHO **(a)** and COH **(b)** formation on CuM clusters.
